# Dynamics of standing deadwood in Austrian forests under varying forest management and climatic conditions

**DOI:** 10.1111/1365-2664.14359

**Published:** 2023-01-24

**Authors:** Janine Oettel, Anita Zolles, Thomas Gschwantner, Katharina Lapin, Georg Kindermann, Karl‐Manfred Schweinzer, Martin M. Gossner, Franz Essl

**Affiliations:** ^1^ Austrian Federal Research Centre for Forests Natural Hazards and Landscape (BFW) Vienna Austria; ^2^ Forest Entomology, Swiss Federal Research Institute of Forest, Snow and Landscape Research (WSL) Birmensdorf Switzerland; ^3^ ETH Zurich, Department of Environmental Systems Science Institute of Terrestrial Ecosystems Zurich Switzerland; ^4^ BioInvasions, Global Change, Macroecology‐Group, Department of Botany and Biodiversity Research University Vienna Vienna Austria

**Keywords:** climate change, deadwood volume, decomposition, forest management, National Forest Inventory, snag persistence, standing deadwood, volume loss

## Abstract

1. Standing deadwood is an important structural component of forest ecosystems. Its occurrence and dynamics influence both carbon fluxes and the availability of habitats for many species. However, deadwood is greatly reduced in managed, and even in many currently unmanaged temperate forests in Europe. To date, few studies have examined how environmental factors, forest management and changing climate affect the availability of standing deadwood and its dynamics.
2. Data from five periods of the Austrian National Forest Inventory (1981–2009) were used to (I) analyse standing deadwood volume in relation to living volume stock, elevation, eco‐region, forest type, ownership and management intensity, (II) investigate the influence of forest ownership and management intensity on snag persistence and (III) define drivers of standing deadwood volume loss for seven tree genera (*Abies*, *Alnus*, *Fagus*, *Larix*, *Picea*, *Pinus* and *Quercus*) using tree‐related, site‐related and climate‐related variables, and predict volume loss under two climate change scenarios.
3. Standing deadwood volume was mainly determined by living volume stock and elevation, resulting in different distributions between eco‐regions. While forest type and management intensity influenced standing deadwood volume only slightly, the latter exhibited a significant effect on persistence. Snag persistence was shorter in intensively managed forests than in extensively managed forests and shorter in private than in public forests.
4. Standing deadwood volume loss was driven by a combination of diameter at breast height, elevation, as well as temperature, precipitation and relative humidity. Volume loss under climate change predictions revealed constant rates for moderate climate change (RCP2.6) by the end of the 21st century. Under severe climate change conditions (RCP8.5), volume loss increased for most tree genera, with *Quercus*, *Alnus* and *Picea* showing different predictions depending on the model used as the baseline scenario. We observed trends towards faster volume loss at higher temperatures and lower elevations and slower volume loss at high precipitation levels. The tree genera most susceptible to climate change were *Pinus* and *Fagus*, while *Abies* was least susceptible.
5. *Synthesis and applications.* We recommend to protect standing dead trees from regular harvesting to ensure the full decomposition process. The consequences for decomposition‐dependent species must be taken into account to evaluate the influences of management and climate change on standing deadwood dynamics.

## Graphical abstract

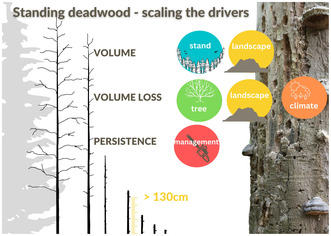

## 1 INTRODUCTION

Deadwood plays a key role in maintaining biodiversity, providing habitats for a plethora of species from various taxonomic groups (Stokland et al., 2012). In addition, deadwood is a key element in processes related to carbon sequestration and emissions in forest ecosystems (Russell et al., 2014; Stokland et al., 2004). As one of the five components of carbon stocks, along with above‐ground and belowground biomass, litter and organic soil, deadwood accounts for about 8% of the current total carbon stock in the world's forests (Pan et al., 2011). Consequently, deadwood has been recognized as an indicator for biodiversity (Lassauce et al., 2011), and also of long‐term carbon storage (Ravindranath & Ostwald, 2008).

In general, deadwood volumes are significantly higher in unmanaged than in managed European temperate forests. Very high deadwood volumes can be reached in forests that have never been silviculturally managed. For instance, Mayer and Neumann (1981) found volumes of 256 m^3^ ha^−1^ in the Rothwald forest reserve in Austria. Unmanaged forests whose management has only recently been abandoned can accumulate deadwood volumes of 50 m^3^ ha^−1^ in European beech‐dominated NFR in Germany and Switzerland (Schall et al., 2021) or 92 m^3^ ha^−1^ in beech‐dominated NFR in Austria (Oettel et al., 2020). Current average volumes in managed forests range from 8 to 20 m^3^ ha^−1^ (Forest Europe, 2015), with most of them remaining far below the threshold of 20–50 m^3^ ha^−1^ proposed by Müller and Bütler (2010) to allow the majority of deadwood‐related species to persist. Accordingly, many saproxylic species in Europe—especially species that depend on large amounts or specific characteristics of deadwood—have declined considerably (Seibold et al., 2015), and their occurrence is often restricted to isolated populations (Lassauce et al., 2011).

Deadwood characteristics include diameter, stage of decomposition and type of deadwood—stumps, standing and lying deadwood, as well as dead branches and dead roots are commonly distinguished (M. E. Harmon et al., 1986)—and are likely to affect the diversity and composition of saproxylic species. In general, saproxylic species assemblages differ depending on the type of deadwood (Ulyshen & Hanula, 2009), with standing deadwood (hereinafter: snags) being of high conservation value for many related species (Abrahamsson & Lindbladh, 2006). Snags in particular provide habitats for lichens (Parisi et al., 2016) as well as nesting or foraging opportunities for many birds (Roberge et al., 2008) and bats (Jung et al., 1999).

However, snags have been shown to be rare in managed forests, but occur also in rather low proportion in many recently unmanaged forests in Central Europe (Christensen et al., 2005; Müller & Bütler, 2010). For example, snags account for 2.5% of total standing volume in managed forests in Austria (Gschwantner, 2019), while their share increases to about 4.8% in NFR (Oettel et al., 2020), and to about 8.9% in primeval forests (Motta et al., 2015). Previous studies have further demonstrated that, in addition to forest management intensity, local forest stand characteristics (Russell et al., 2012), climate (Oberle et al., 2018), mortality factors (Hararuk et al., 2020) and species‐specific wood properties (Petrillo et al., 2016) influence snag availability by substantially affecting their decomposition.

Snag decomposition is a complex process defined as a combination of biological respiration, leaching and fragmentation (M. E. Harmon et al., 1986; Russell et al., 2015) and is primarily driven by saprotrophic organismic activity (Kahl et al., 2017). Harmon et al. (1986) further specified that fragmentation, also described as mechanical degradation (Ulyshen, 2016), refers to a decrease in wood density and volume. The need to quantify temporal changes in snag decomposition patterns has increased due to changing climatic conditions and their uncertain consequences (Chagnon et al., 2022; Herrmann & Bauhus, 2013). Fraver et al. (2013) pointed out that decomposition rates, commonly derived from reductions in wood density over time, underestimate rate loss as they do not account for volume loss over time. However, consideration of volume loss complements characterization of snag decomposition patterns and has important practical applications (Müller‐Using & Bartsch, 2009).

Generally, snag decomposition is known to be affected by bark cover and, accordingly, the presence, diversity and activity of dead wood‐depending biota (Ulyshen, 2016). Besides this, climatic (Herrmann & Bauhus, 2013; Russell et al., 2015) and site‐related variables (Fravolini et al., 2018; Herrmann et al., 2015; Shorohova & Kapitsa, 2014) impact snag decomposition dynamics as well, leading to variations in snag persistence. Snag persistence refers to the time between the death of a tree and its fall (Oberle et al., 2018). Both the persistence and the decomposition behaviour of snags influence forest biodiversity (Crecente‐Campo et al., 2016; Přívětivý et al., 2018) as well as the carbon storage (Fravolini et al., 2018; Freschet et al., 2012).

So far, only few studies have simultaneously assessed the influence of site‐related variables, forest management, and changing climatic conditions on snag availability, persistence and decomposition (Přívětivý et al., 2016, 2018; Vrška et al., 2015). Therefore, scientists and conservationists have repeatedly emphasized the urgent need to assess snag dynamics in the face of climate change to develop efficient management and conservation strategies (e.g. Aakala et al., 2008; Bouget et al., 2014; Brunet & Isacsson, 2009). The limited ability to extrapolate from local monitoring projects and case studies due to low spatial and temporal coverage (Levrel et al., 2010) means that National Forest Inventories (NFIs) play a crucial role in terms of aggregating information on snags at large scales and thus in predicting spatiotemporal changes in response to global change drivers (Rondeux & Sanchez, 2010).

Here, we investigated the dynamics of snags using data from five survey periods of the Austrian NFI (1981–2009). Specifically, we analysed snag volume in relation to forest types, elevation, ownership (private vs. public) and management intensity; and snag persistence based on individuals that died and fell during the observation period. Using volume loss over time, we defined drivers of snag decomposition and predicted volume loss under two climate change scenarios. We hypothesized (I) snag volume to vary among forest types, being higher in mountainous compared to lowland eco‐regions and being low under intensive forest management, (II) forest ownership and management intensity to influence the snag persistence, with the latter being significantly lower in intensively managed forests than in extensively managed forests, (III) higher temperature and relative humidity to accelerate the volume loss of snags and (IV) the rate of volume loss to increase with advancing climate change.

To the best of our knowledge, this is the first study predicting climate change impact on snag volume loss in temperate European forests. Achieving the defined objectives will help to provide urgently needed baseline information for evidence‐based decision‐making regarding snag management and thus forest biodiversity conservation and carbon cycling under climate change.

## 2 MATERIALS AND METHODS

### 2.1 Study area

Approximately 50% of the land surface (4.02 million ha) of Austria is covered by forests (Russ, 2019). The majority of Austrian forests (82%) are privately owned: 54% are considered small (less than 200 hectares), 10% are medium‐sized (200–1000 ha) and 18% are considered large‐scale (more than 1000 ha) private property. The remaining 18% of forests are publicly owned: 15% by the Austrian Federal forests (ÖBf), 2% by municipalities and 1% by the federal states (BFW, 2019b).

Forest distribution in Austria is characterized by a large elevational range (120–2100 m a.s.l.) and comprises a variety of forest types. The main tree species based on their share in growing stock are Norway spruce (*Picea abies* Karst.) (60.4%), European beech (*Fagus sylvatica* L.) (10.0%), European larch (*Larix decidua* Mill.) (6.6%), Scots pine (*Pinus sylvestris* L.) (6.2%) and silver fir (*Abies alba* Mill.) (4.4%). The remaining share is accounted for by other coniferous (1.3%) and broadleaved (11.0%) species (BFW, 2019a).

### 2.2 NFI data

#### 2.2.1 Site‐specific and forest management–related data

Data from the third (NFI 3, 1981–1985) to the seventh (NFI 7, 2007–2009) Austrian NFI were used. We did not include data from the first and second NFI because temporary plots were used for which repeated measurements are not available. The Austrian NFI provides a network of permanent sample plots, arranged at the corners of square‐shaped clusters with a side‐length of 200 m. The clusters are located at the intersection points of a systematic 3.89 km × 3.89 km grid. The total grid consists of 5582 clusters and therefore 22,236 sample plots, about half of which are located on forested land (Gschwantner et al., 2010).

For further analysis, plots located completely on forest land and with the presence of trees were included. In doing so, we excluded plots from the analysis for which the type of land cover was ambiguous and therefore could not be clearly assigned to forest. At each sample plot, stand‐, site‐ and management‐related variables are assessed during the field measurements of each survey period on a circular fixed‐area plot of 300 m^2^ size (Gschwantner et al., 2010). Each plot is categorized in terms of eco‐region following Kilian et al. (1994), forest type based on the current stand‐characterizing tree species and crown coverage of stand closure classes. Five different categories of forest ownership are distinguished, and the intensity of forest management is assessed at the plots as well. The distinction between intensive and extensive forest management is based on classification criteria (e.g. signs of management activities like the presence and age of stumps, along with the accessibility and steepness of the terrain) as specified in the field manual of the Austrian NFI (Hauk & Schadauer, 2016). The site‐specific variables elevation, aspect and slope are also recorded during the NFI surveys. All categories are described and summarized in Table S1 following the Austrian NFI manual (Hauk & Schadauer, 2016).

#### 2.2.2 Standing deadwood data

The angle‐count sampling method according to Bitterlich (1948, 1984) was applied for trees with a minimum threshold diameter at breast height (dbh) of 10.5 cm and by using a fixed angle of sight (1:25, referring to a basal area factor of four). Trees with a dbh of less than 10.5 cm but above or equal to 5.0 cm were assessed on circular sample plots with a size of 21.2 m^2^. For each living and dead sample tree, the tree species, dbh [mm], and position within the plot (distance [cm] and azimuth [gon]) were recorded. If a tree was newly broken during the observation period, this was noted in each survey. Tree heights [dm] were measured for the complete sample during the third and seventh NFI and for every fifth sample tree during the fourth to sixth NFI. A distribution of snag dbh and height values for the most recent survey (seventh NFI) are provided in Figure S1. The heights of unmeasured trees were obtained using data models (Gabler & Schadauer, 2008). These data models rely on the dbh and height of the previous NFI at time 1 and the dbh of the following NFI at time 2. Measuring tree heights on a sub‐sample in field data collection is widely used in NFIs to save cost and time, since the aim of NFIs is to provide statistical data and the basis for sustainable forest utilization and forest policy. As shown by Puletti et al. (2019) and Hekkala et al. (2016), using modelled heights for snag volume calculations is widely accepted.

### 2.3 Data processing and analysis

For further analysis, we considered snags as entire standing dead trees with a minimum height of 1.3 m and a minimum dbh of 5.0 cm. Stumps with a height of less than 1.3 m were not included. Three datasets were prepared. Dataset 1 was prepared for snag volume analysis as repeated measures per plot, while dataset 2 (for snag persistence analysis) and dataset 3 (for fragmentation analysis) were prepared on a per tree basis with repeated measures and included stand‐, site‐ and management‐related variables from the most recent tree observation. All analyses were performed in the R environment version 3.6.2 (R Core Team, 2019) using the packages maps, plotrix, rstatix, ggpubr and ggplot2. Model scripts were written in Python 3.9.1 (Python Software Foundation, 2020).

#### 2.3.1 Standing deadwood volume

**FIGURE 1:**
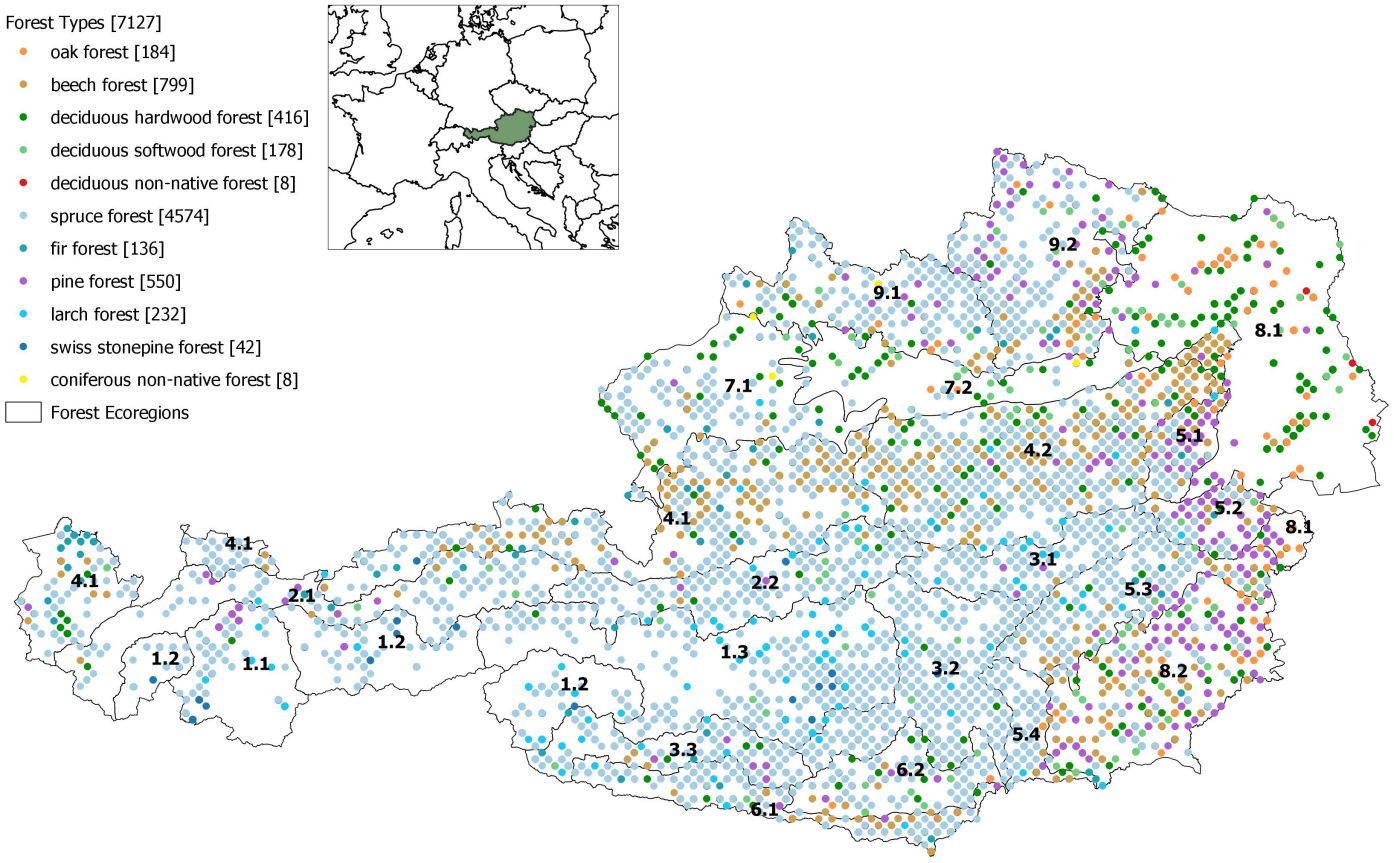
Map of Austria including eco‐regions (according to Kilian et al. (1994) and described in Table S2) and the Austrian NFI plots located entirely in forests. Different colours indicate the respective forest type based on the current stand‐characterizing tree species. All forest types are presented, resulting in a total number of 7127 plots.

The stem volume estimates for individual snags with a minimum dbh of 5.0 cm were obtained using the same volume models employed for living trees described by Gabler and Schadauer (2008). The distribution of NFI plots (*n* = 7127) classified by forest types based on the current stand‐characterizing tree species in Austria is illustrated in Figure 1. The ecoregions according to Kilian et al., 1994 summarize landscapes with largely uniform climatic characteristics and site‐typical environmental factors. A summary of ecoregions is provided in Table S2. We excluded the most infrequent forest types, that is, Swiss stone pine forest (*n* = 42), coniferous non‐native forest (*n* = 8) and deciduous non‐native forest (*n* = 8) resulting in a dataset 1 of 7069 sample plots observed over five NFI periods. A summary of the living and snag volumes per forest type is provided in Table 1.

**TABLE 1:** Summary of living (Vliving) and snag (Vsnag) volume (m^3^ ha^−1^) including mean, standard deviation (SD), minimum and maximum values for the different forest types (dataset 1).

Forest type	Nr of plots	Volume (m^3^ ha^−1^)	Mean	SD	Min	Max
Beech forest	799	Vliving	337.03	206.40	0.00	1349.48
		Vsnag	10.50	23.66	0.00	208.34
Deciduous hardwood forest	416	Vliving	206.01	150.29	0.00	836.91
		Vsnag	6.35	17.56	0.00	129.32
Deciduous softwood forest	178	Vliving	218.33	185.89	0.00	1241.13
		Vsnag	6.85	18.72	0.00	111.23
Fir forest	136	Vliving	493.85	259.23	0.00	1626.89
		Vsnag	11.32	24.75	0.00	123.47
Larch forest	232	Vliving	273.01	191.63	0.00	870.68
		Vsnag	7.62	16.75	0.00	94.80
Oak forest	184	Vliving	232.49	153.68	0.00	728.37
		Vsnag	6.93	17.54	0.00	109.83
Pine forest	550	Vliving	364.19	183.09	0.00	1111.69
		Vsnag	7.06	18.74	0.00	144.55
Spruce forest	4574	Vliving	369.68	236.01	0.00	1494.60
		Vsnag	9.88	28.44	0.00	508.13

We modelled snag volume using (1) living volume, (2) elevation, (3) eco‐region, (4) forest type, (5) forest ownership, (6) forest management intensity and (7) NFI survey period as predictors. The distribution of snag volume is characterized by zero inflation and truncation, as negative values are not possible. We therefore chose a Random Forest (RF) model based on uncorrelated classification and regression trees. RF models are able to adapt to complex, nonlinear relationships between variables and deal with interactions between variables (Elith et al., 2008). On the other hand, they are quite sensitive to the quality of the input data in terms of representativeness and to extrapolation (Hengl et al., 2018).

Since forest type, ownership and management intensity are categorical, non‐hierarchical variables, we used one‐hot encoding for their consideration in the model. This method creates different Boolean variables indicating whether or not a particular category was present for an observation. On the basis of an even distribution of sample plots, we formed seven categories of deadwood volume (see Table S3). A random set of sample plots was selected from each category, with the number of plots selected being equal to the number of plots from the smallest category. This procedure ensured equal consideration of different deadwood volumes in the model. For further analysis, however, we used absolute values. In building the model, we used a training dataset (80%) by randomly selecting samples from the seven categories. For the test dataset, we used the remaining 20% of data. To determine the best scoring selection of variables and to fit the model, we used recursive feature elimination (RFE). RFE also eliminates dependencies and collinearities among predictors (Darst et al., 2018). We used the resulting predictor ranking to build a set of models with varying numbers of predictors. To evaluate the performance of each model, we compared *R* ^2^ values based on our test data. In the final model, the predictors were ranked according to their relative importance (Gini importance), which is the sum over the number of splits including the predictor, proportional to the number of samples it splits (Nembrini et al., 2018).

#### 2.3.2 Standing deadwood persistence

Snag persistence was calculated as time between the death of each individual tree and its fall. The average year between two NFI survey periods was assumed as time of death, that is, if a tree was recorded as alive in 1984 (NFI 3) and dead in the next survey in 1990 (NFI 4), we assume time of death was in 1987. In case of snag fall during the observation period (1981–2009), the underlying cause was classified as management‐induced (managed) or naturally induced (natural) fall. We included only individuals that died and fell during the observation period (1981–2009). To investigate the influence of forest owners' behaviour and management practices in relation to snags, individuals which died before the observation period or did not fall during the observation period had to be excluded. Dataset 2 thus consists of 577 individuals.

As data were non‐normally distributed, we used the Kruskal–Wallis nonparametric test (Kruskal & Wallis, 1952) to test for differences in snag persistence among the categories: (I) reason for snag fall, (II) forest management intensity and (III) forest ownership. To account for significant influence among individual forest ownership categories, Dunn's multiple comparison test (Dunn, 1961) was applied as post‐hoc test.

#### 2.3.3 Standing deadwood volume loss

Volume loss of snags was estimated in terms of volume loss rates during a specified time period and expressed as a constant *k*_loss_. We calculated *k*_loss_ for all snags based on the volume loss over time for each snag:

(1) $$k_{\text{loss}}=\frac{\left(V_{0}-V_{t}\right)}{t},$$

where *V*_0_ is the volume (in m^3^) at the time of death, *V*_*t*_ is the volume (in m^3^) at the time of the last measurement before snag fall and *t* is the time period in years. *V*_0_ was calculated as the mean value between the preceding and following NFI survey periods to assume the time of death. We used volume rather than converted biomass to calculate loss rates as it allows for easier interpretation and application by forest management. To enable calculation of volume loss, only snags that occurred in at least two of the NFI survey periods were considered, resulting in a total number of 5331 trees in dataset 3. Table 2 provides an overview of the distribution of diameter (in mm) and volume (in m^3^) per tree genus.

**TABLE 2:** Summary of snag dbh (in mm) and volume (m^3^) including mean, standard deviation (SD), minimum and maximum values for the different tree genus investigated in this analysis (dataset 3). Only snags that occurred in at least two of the NFI survey periods were considered

Tree genus	Nr of snags	Variable	Mean	SD	Min	Max
Abies	318	dbh (mm)	282.60	168.29	51.00	804.00
		V (m^3^)	0.90	1.17	0.01	7.08
Alnus	116	dbh (mm)	149.80	66.95	50.00	393.00
		V (m^3^)	0.14	0.16	0.01	1.08
Fagus	243	dbh (mm)	176.80	126.07	50.00	691.00
		V (m^3^)	0.33	0.60	0.01	5.10
Larix	450	dbh (mm)	206.90	143.38	49.00	1205.00
		V (m^3^)	0.43	0.89	0.01	14.63
Picea	3569	dbh (mm)	172.40	137.80	43.00	1339.00
		V (m^3^)	0.35	0.83	0.00	19.90
Pinus	542	dbh (mm)	208.70	110.50	50.00	640.00
		V (m^3^)	0.38	0.43	0.01	3.47
Quercus	93	dbh (mm)	249.30	167.04	55.00	978.00
		V (m^3^)	0.63	1.15	0.01	8.64

Depending on the snag persistence of individuals, we were able to provide separate volume loss rates per tree along the decomposition process. The *k*_loss_‐values were expressed in year^−1^ units. We chose this linear model after checking the relationship between final and initial volume in our dataset, which showed a strong linear relationship across all tree genera (see Figure S2). For all investigated tree species, objects with negative volume loss rates were observed—a finding that has already been reported previously for deadwood decomposition rates (Kahl et al., 2017). In our dataset, 43% of the observations displayed negative rates, indicating a volume increase, which is rather unlikely. However, 82% of negative rates are neglectable with values above −0.01. Increases in volume increment may be the result of an increase in wood moisture content or lifting of the bark during the decomposition process. Seasonal variation in temperature or moisture from one NFI survey to the next is also likely, which may have resulted in diameter variation. Although this is an important methodological issue, our data show that different tree species are affected by this issue to a similar extend, meaning that the relative ratio of volume loss rates between tree species remained largely unbiased (see Figure S3). We additionally performed sensitivity analyses to test the influence of these negative values on our results (see below).

To model volume loss of the seven main tree species (assessed at genus level: *Picea, Fagus, Abies, Pinus, Quercus, Larix, Alnus*), we considered eight potential explanatory variables. These were grouped into the tree‐related variable (I) dbh, the stand‐related variable, (II) crown cover, the site‐related variables (III) elevation, (IV) slope and (V) aspect, and the climate‐related variables (VI) temperature, (VII) precipitation, and (VIII) relative humidity (see Table S1). Annual mean values for temperature and relative humidity as well as annual sums for precipitation were derived from daily measurements (1981–2009) taken by weather stations of the Central Institution for Meteorology and Geodynamics (ZAMG, 2020) and interpolated for NFI plots. The dbh, crown cover and climate‐related variables were taken from the most recent survey period for each snag, as we assumed these to have the greatest influence on the most recent measurement.

RF regression modelling was used to analyse the relationship between the volume loss constant *k*_loss_ and the explanatory variables. The overall dataset was split into a training and a test dataset. The training dataset was created by randomly selecting samples (80%) from the overall dataset. We performed modelling with different input datasets to assess whether and how negative volume loss rates and broken tree individuals affect model performance:

model a1: positive and negative volume loss *k*_loss_ (all).

model a2: positive and negative volume loss *k*_loss_ (all), excluding broken individuals.

model b1: only positive volume loss *k*_loss_.

model b2: only positive volume loss *k*_loss_, excluding broken individuals.

We compared model performance based on the main occurring coniferous and deciduous tree genus *Picea* and *Fagus*. A pairwise comparison of models a1‐a2, b1‐b2 indicated better performance when excluding broken individuals (a1, b1). The two remaining models exhibited also lower variation in performance for both *Picea* (*R* ^2^ = 0.79–0.99) and *Fagus* (*R* ^2^ = 0.75–0.87). We thus decided to proceed with models a2 and b2 (all models for *Fagus* and *Picea* are provided in Table S4). Datasets 3 selected for volume loss analyses consist of 5331 and 2694 samples, respectively.

#### 2.3.4 Climate change scenarios

To predict future climate impact on volume loss, we used two Representative Concentration Pathways climate change scenarios (IPCC, 2014; Moss et al., 2010). We chose these two scenarios because they cover the entire range from moderate (RCP2.6) to severe climate change scenarios (RCP8.5). RCP2.6 will keep global warming likely below 2°C above pre‐industrial temperatures by the end of the 21st century (Van Vuuren et al., 2007), while RCP8.5 predicts global mean surface temperature increases of 3.7°C (IPCC, 2014). Mean precipitation in Austria is projected to decrease in summer and increase in winter (Kromp‐Kolb et al., 2014), resulting in a decrease in relative humidity.

We used daily mean temperatures, daily precipitation and daily relative humidity from the global climate model (GCM) MPI‐ESM‐LR (Max Planck Institute for Meteorology, Germany), dynamically downscaled to the regional level (spatial resolution of 0.11°, ~12.5 km cell size) using REMO2009 r1i1p1. Temperature and precipitation were bias corrected by Switanek et al. (2017). Data for temperature and precipitation were downloaded from the CCCA data server (https://data.ccca.ac.at) and data for relative humidity from the Cordex portal (http://www.euro‐cordex.net/) for the period from 2000 to 2100.

The RF models a2 and b2 forced by the observed data were used as baseline conditions for climate projection of species‐wise volume loss development. All climate model variables were interpolated for the NFI plots and summarized to annual means of temperature and relative humidity as well as annual sums of precipitation. In addition, we used elevation to predict the volume loss of snags. Since dbh is an essential variable in our model, we compared *k*_*loss*_‐values for three exemplary dbh values: 100 mm, 200 mm 300 mm, each for the period from 2000 to 2100.

## 3 RESULTS

### 3.1 Drivers of snag volume

**FIGURE 2:**
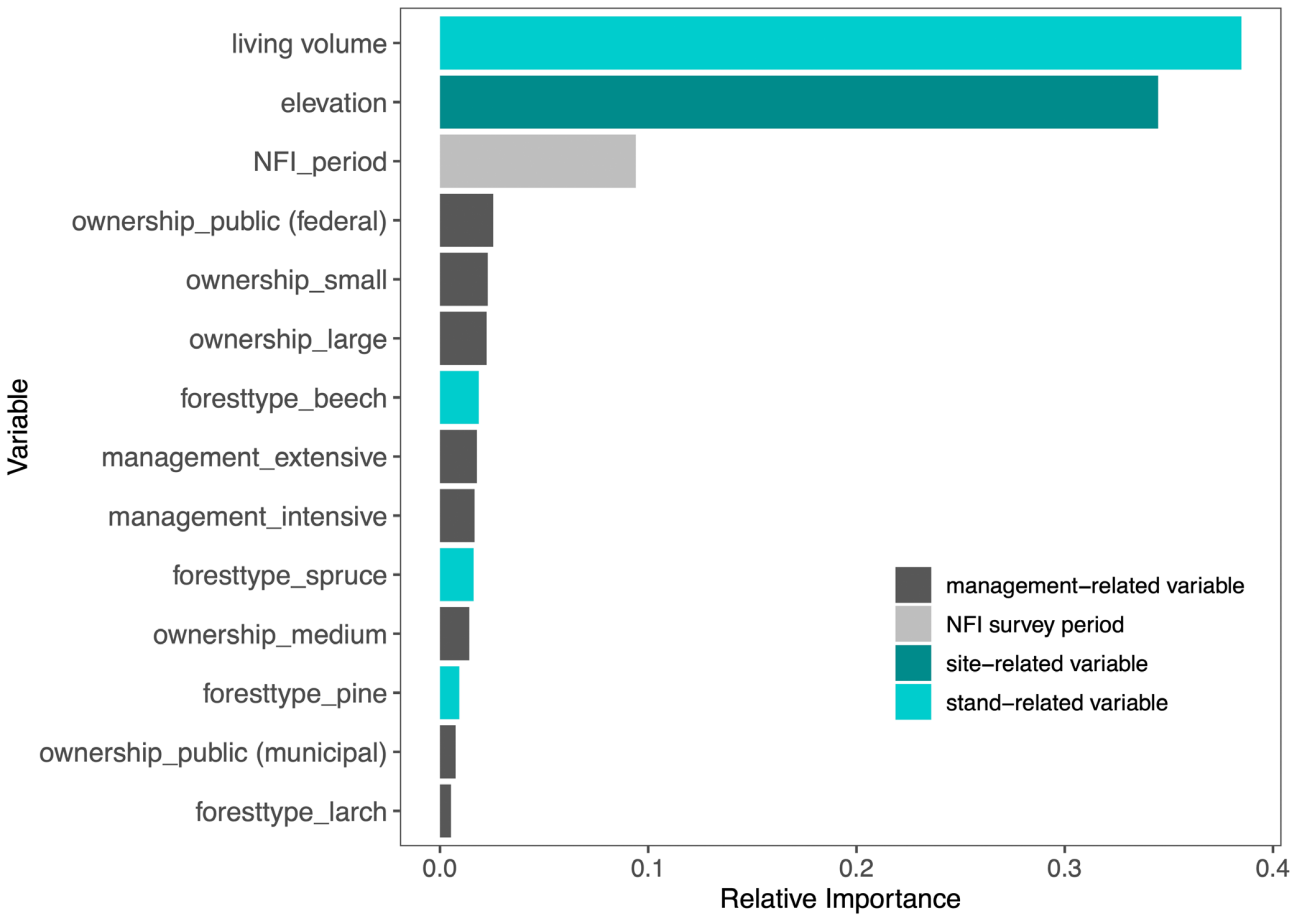
Relative importance of the explanatory variables in the Random Forest regression model for snag volume. Different colours indicate categories of variables. For definitions of variables, see Table S1.

The RF model explained 40.2% of the variance of snag volume and RFE selected 14 variables to fit the model (Figure 2). For snag volume, the stand‐related and site‐related parameters living volume (38.4%) and elevation (34.4%) were of the greatest relative importance. In contrast to our hypothesis (H I), management‐related parameters, such as forest ownership (0.7%–2.5%), management intensity (1.4%–1.6%), as well as forest type—that is, spruce (1.6%) and beech‐dominated forest types (1.8%)—were of minor relevance. To obtain information on the trend and magnitude of the relationship between snag volume and the influencing predictors, we generated linear regression plots for continuous variables and boxplots for categorical variables based on the observations (see Figure S4a–f).

**FIGURE 3:**
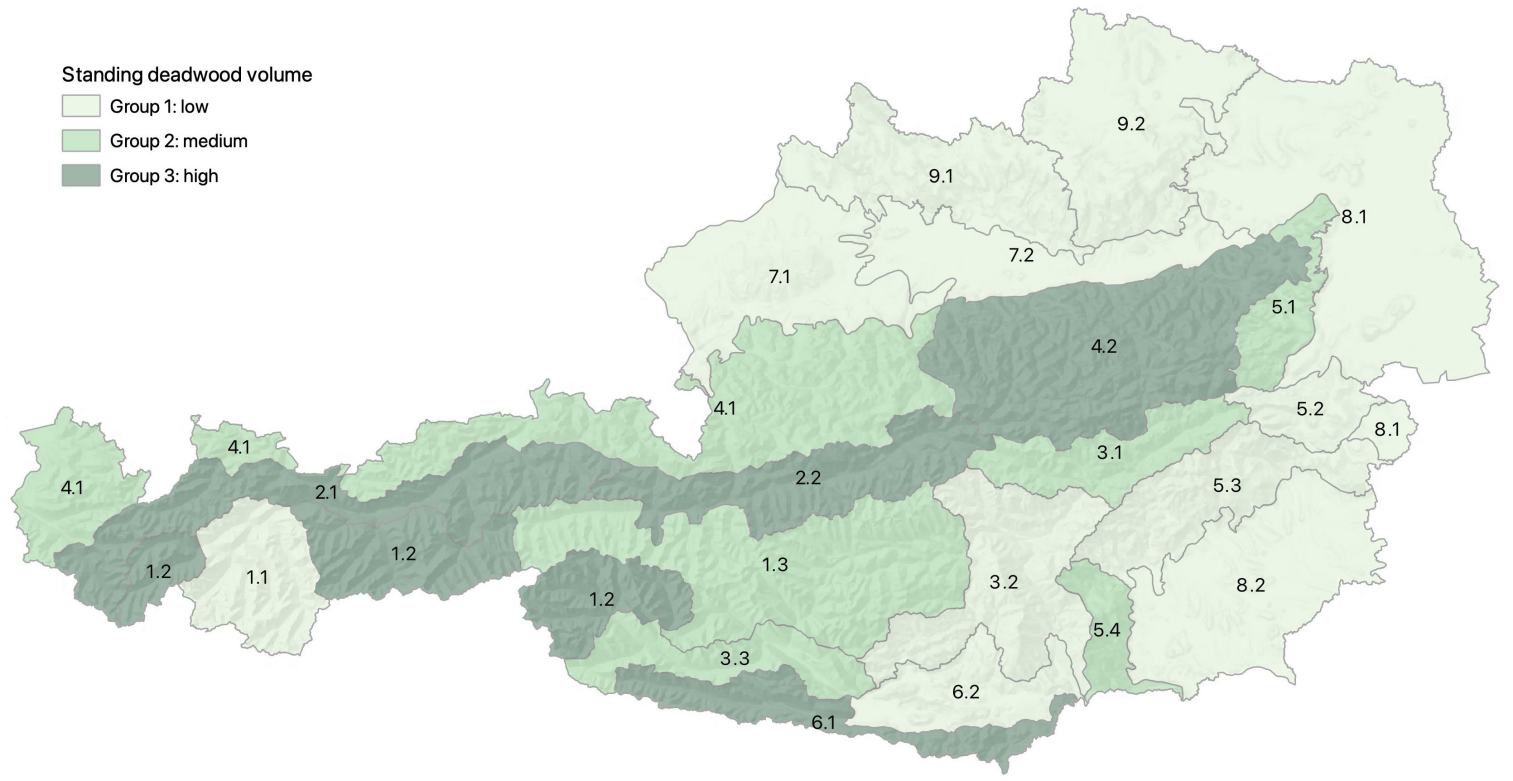
Map of snag volume distribution based on the Austrian National Forest Inventory (NFI) in Different eco‐regions (see Table S1 for a description of eco‐regions). The mean snag volume was summarized as low (4–8 m^3^ ha^−1^), medium (8–12 m^3^ ha^−1^) and high (12–16 m^3^ ha^−1^), representing the snag volume in managed forests of the different eco‐regions.

We found different regional patterns in snag volume, ranging from mean values of 4.00 m^3^ ha^−1^ in the Bohemian Massif region (eco‐region 9.1, see Table S1) to 15.42 m^3^ ha^−1^ in the inner alpine region (eco‐region 1.2, see Table S1). Eco‐regions were summarized to three groups based on their mean values (Figure 3): Group 1 represents eco‐regions with low snag volumes (mean: 4–8 m^3^ ha^−1^) including 11 out of the 22 eco‐regions; Group 2 represents moderate volumes (mean: 8–12 m^3^ ha^−1^) and consists of 6 eco‐regions; Group 3 represents high volumes (mean: 12–16 m^3^ ha^−1^) and consists of 5 eco‐regions.

### 3.2 Effects of forest management on snag persistence

**FIGURE 4:**
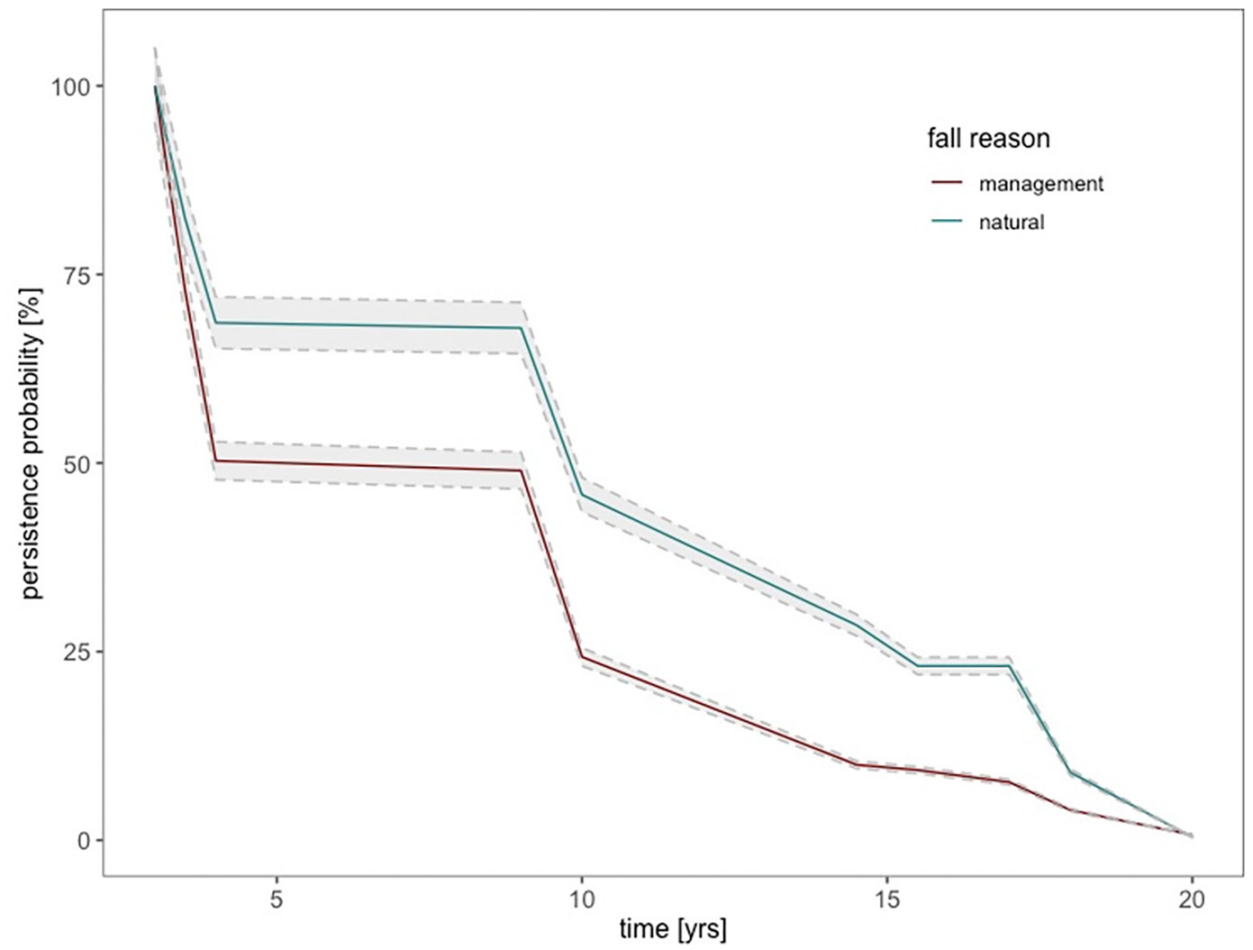
Snag persistence probability [%] over time (in years) by fall reason, either natural (blue, *n* = 277) or by management (red, *n* = 300), including 95% confidence interval (grey). Snag persistence was calculated as time between the death of each individual tree and it's fall over 5 periods of Austrian National Forest Inventory (1981–2009). The mean snag persistence was significantly higher (*p* < 0.001) for natural (9.5 years) compared to management‐induced (7.1 years) snag fall.

Snag persistence was investigated for 577 individuals, with 277 falling naturally and 300 falling due to management. Overall mean persistence was 5 years with a range of 3–20 years. The snag persistence was significantly higher (*p* < 0.001) for natural (9.5 years) compared to management‐induced (7.1 years) snag fall, as shown in Figure 4.

**FIGURE 5:**
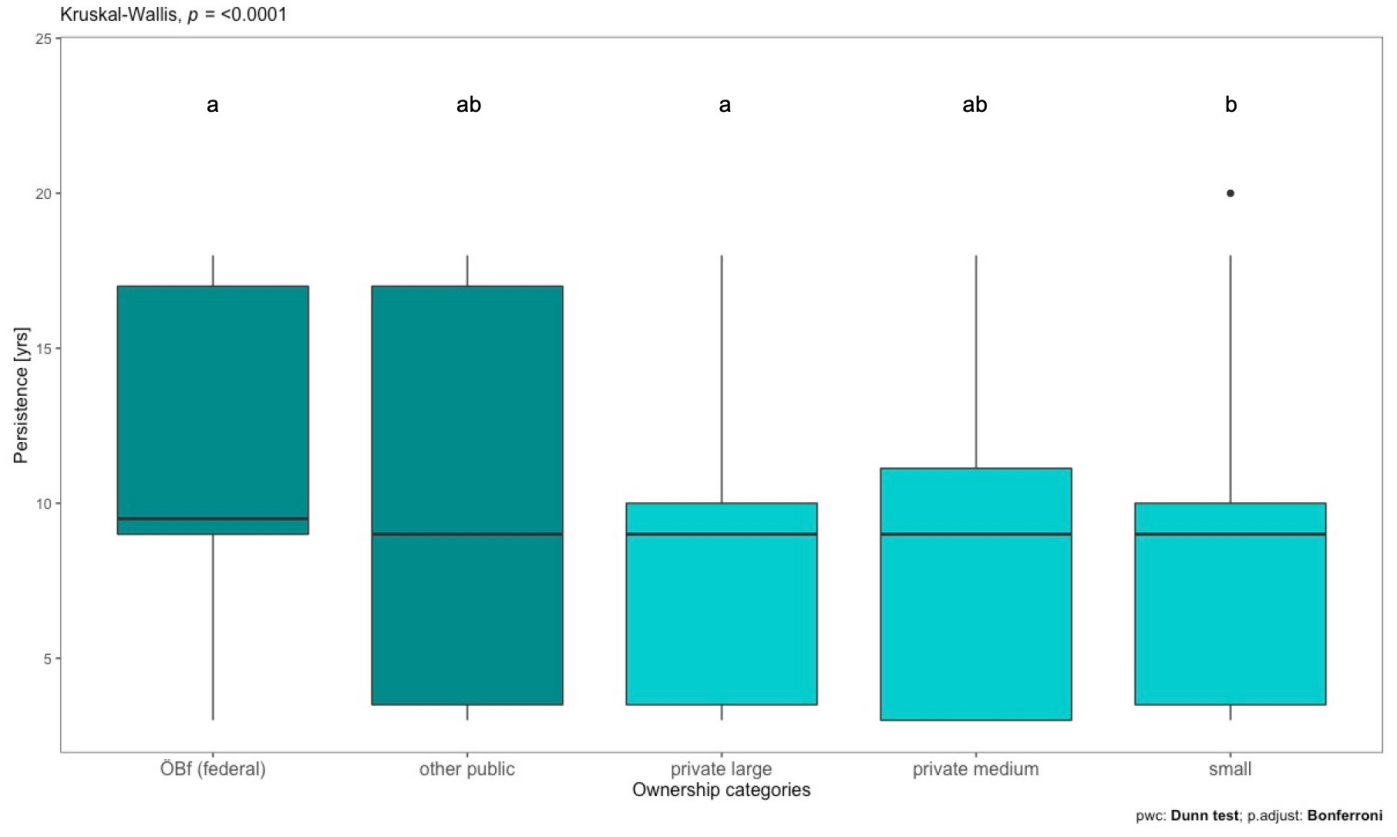
Snag persistence (in years) for five categories of forest ownership: publicly owned forests (dark blue) are divided into Austrian Federal Forests (ÖBf) and other public forests; privately owned forests (light blue) are divided into large (area > 1000 ha) and medium‐sized (200 ha > area ≤ 1000 ha) forests, while all ownership types are tallied together for small scale forests (area ≤ 200 ha). Median values, 25% and 75% percentiles (box) and min‐max values (whiskers) are shown and the results of the Kruskal–Wallis test and pairwise comparisons based on post‐hoc tests (Dunn test) with related statistical components are added. Different letters indicate statistically significant differences at *p* < 0.05. Bars with no common letters are significantly different (*p* < 0.05).

As hypothesized (HII), snags fell after a shorter period of time (mean of 7.7 years) in intensively managed forests than in extensively managed forests (mean of 9.9 years; *p* < 0.001). Snag persistence also differed among ownership categories. Pairwise comparisons based on post‐hoc tests revealed that the snag persistence differed significantly between small private forests and the publicly owned Austrian Federal forests (ÖBf) (*p* < 0.001; Figure 5).

### 3.3 Modelling volume loss of standing deadwood

**FIGURE 6:**
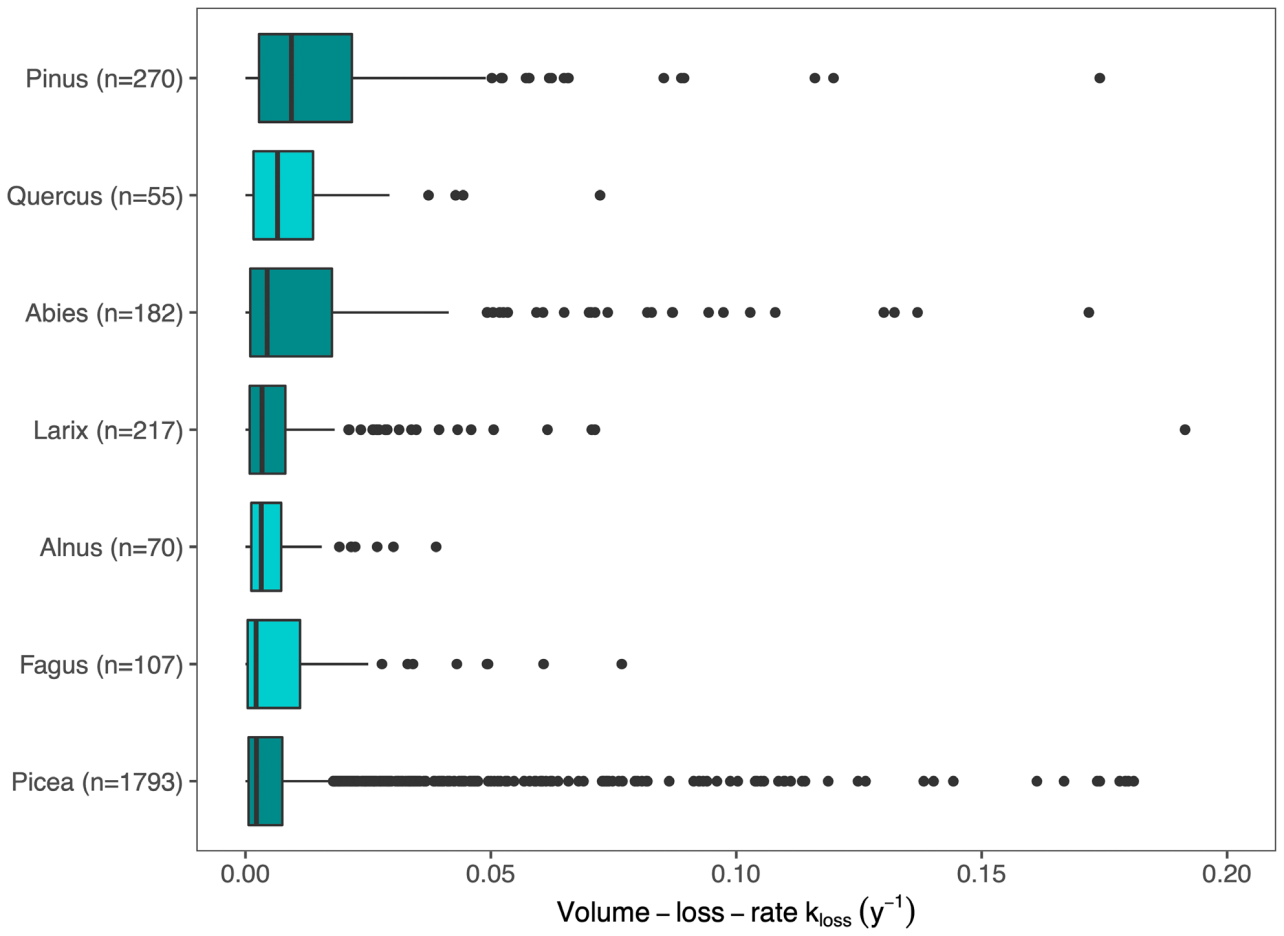
Volume loss rate *k*_loss_ for snags of seven tree genera over 5 survey periods of the Austrian NFI (1981–2009). Median values, 25% and 75% percentiles (boxes), min‐max values (whiskers) and outliers are presented. Tree species are ordered by decreasing median values. The coloration of coniferous (dark blue) and deciduous tree species (light blue) is for illustration only.

The investigated tree species exhibited distinct volume loss with the highest *k*_loss_‐rates (calculated as mean ± standard deviation (SD); excl. negative values) determined for *Pinus* (0.016 ± 0.021 year^−1^) and the lowest for *Picea* (0.010 ± 0.081 year^−1^; Figure 6). Among the deciduous species, *Quercus* showed highest fragmentation rates (0.011 ± 0.014 year^−1^) and *Fagus* (0.010 ± 0.027 year^−1^) the lowest.

In the a2 and b2 models, the variables dbh, elevation, temperature, precipitation and relative humidity were the best predictors for snag fragmentation. Aspect, slope and crown cover were not considered in the final models. Variable importance calculated from the RF algorithm allowed the variables to be ranked according to their relative contribution to the final models (see Table 3). In both models, the ranking clearly highlighted the importance of dbh for all tree genera (in model a2: 32%–81%, in model b2: 55%–69%), and according to our hypothesis (H III), the importance of temperature for all coniferous species as well as *Alnus* (11%–22%), and of relative humidity for all species (12%–23%), except *Larix* and *Quercus*. With the reduced dataset of model b2, a shift in variable ranking was observable for *Abies, Picea* and *Quercus*. *Abies* model b2 showed an increased importance of elevation (12%) along with a decreased importance of temperature (10%). A similar trend towards an increased importance of elevation was also observed in the *Picea* model b2, while the importance of temperature and relative humidity decreased. The opposite was found for *Quercus*, resulting in a higher importance of temperature and relative humidity (10%–14%) and a lower importance of elevation (4%).

**TABLE 3:** Significant variables, their ranking and relative importance within the random forest regression models a2 (positive and negative volume loss rates *k*_loss_, excluding broken individuals) and models b2 (only positive volume loss rates *k*_loss_, excluding broken individuals) for volume loss

Variable	*Abies*	*Alnus*	*Fagus*	*Larix*	*Picea*	*Pinus*	*Quercus*
**Model a2**							
Elevation	0.09	0.10	0.10	0.09	0.08	0.13	0.06
dbh	0.32	0.45	0.67	0.67	0.61	0.41	0.81
Temperature	0.22	0.18	0.06	0.11	0.11	0.18	0.05
Precipitation	0.14	0.12	0.04	0.06	0.08	0.14	0.03
Relative humidity	0.23	0.15	0.14	0.07	0.12	0.14	0.05
***R*** ^**2**^	**0.71**	**0.82**	**0.79**	**0.70**	**0.87**	**0.80**	**0.58**
**Model b2**							
Elevation	0.12	0.07	0.14	0.11	0.09	0.04	0.04
dbh	0.55	0.56	0.61	0.55	0.69	0.69	0.67
Temperature	0.10	0.20	0.06	0.16	0.08	0.09	0.10
Precipitation	0.09	0.05	0.02	0.09	0.06	0.08	0.05
Relative humidity	0.15	0.12	0.17	0.09	0.08	0.09	0.14
***R*** ^**2**^	**0.95**	**0.99**	**0.99**	**0.97**	**0.75**	**0.96**	**0.95**

Overall, the b2 models (*R* ^2^: 0.75–0.99) showed a better predictive power compared to the a2 models (*R* ^2^: 0.58–0.87). Details on model performances are provided in Figures S5a and S5b. To obtain information on the trend and magnitude of the relationship between snag decomposition constant *k*_loss_ and the influencing predictors, we additionally performed correlations for each tree genus based on the observations for the a2 models (s. Table S5a) and the b2 models (see Table S5b).

### 3.4 Climate change impact on snag volume loss

**FIGURE 7:**
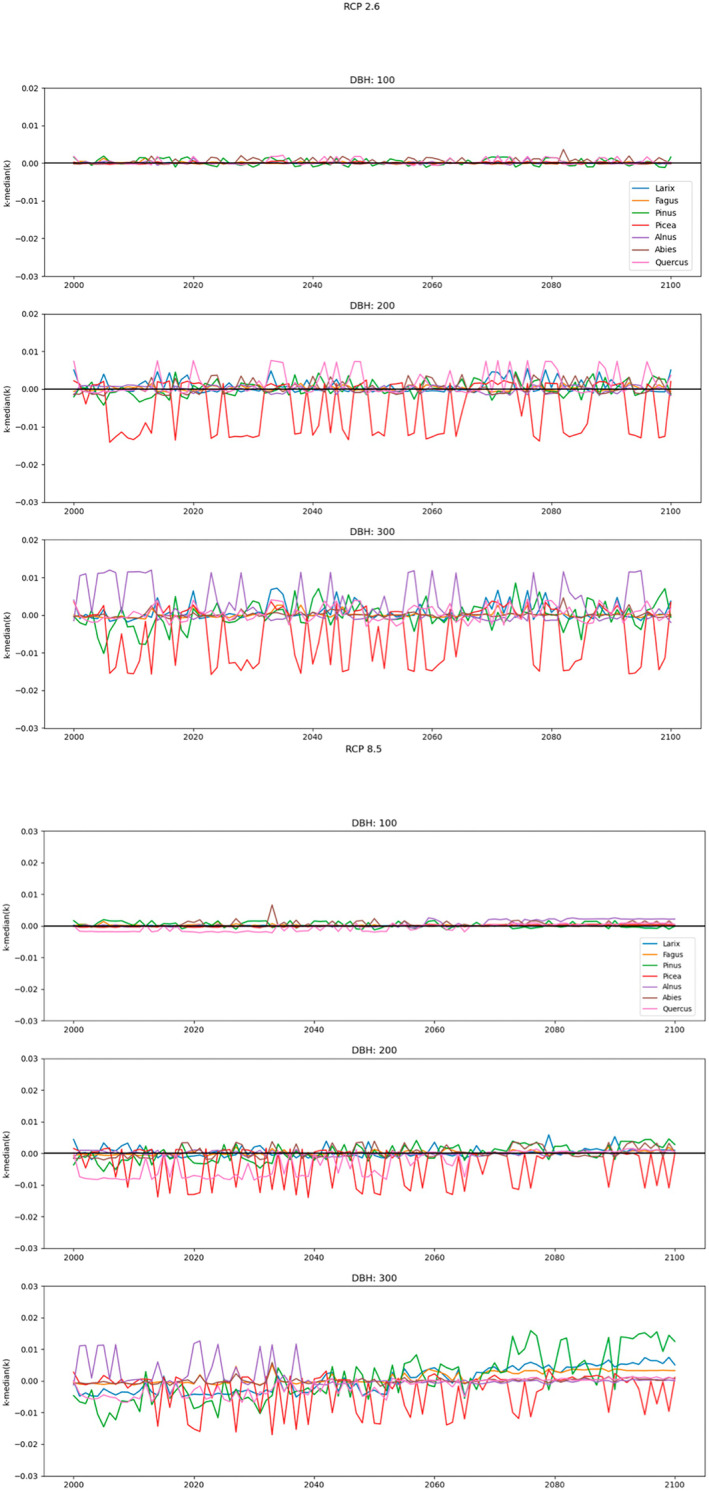
Prediction of snag volume loss rates *k*_loss_ [year^−1^] using **model b2** as baseline condition under climate change scenarios RCP2.6 and RCP8.5 for the seven investigated tree genera (predictions using model a2 as baseline condition are provided in Figure S6 and Table S6). Subplots refer to different tree diameters at breast height (dbh: 100, 200, 300 mm). For comparison of volume loss among the investigated tree genera, the *k*_loss_‐rates are expressed as deviations from the respective median. The slopes and averages of the individual tree genera per diameter at breast height (dbh) are provided in Table 4.

Our predictions for model b2 revealed constant *k*_loss_‐values under moderate climate change for all tree species and tree diameters when comparing means of the first and the second half of the 21st century. Under severe climate change, the *k*_loss_‐values increased, as hypothesized (H IV), for small‐diameter *Alnus* (0.01), large‐diameter *Larix* (0.02), and medium‐ and large‐diameter *Fagus* (0.01–0.02), *Quercus* (0.02) and *Pinus* (0.03–0.04) (Figure 7, Table 4). *Abies* and *Picea* were least sensitive to climate change, showing no apparent trend. In general, the increase in *k*_loss_‐values was more pronounced towards the end of the prediction period (2060–2100).

**TABLE 4:** Slopes and averages of *k*_loss_‐rates using model b2 as baseline condition for the seven investigated tree genera per diameter at breast height (dbh: 100, 200, 300 mm) under contrasting climate change scenarios until the end of the 21st century (slopes and averages using model a2 as baseline condition are provided in Figure S6 and Table S6)

Tree genus	dbh	RCP2.6		RCP8.5	
		Slope	Average	Slope	Average
*Abies*	100	2.59++E‐06	2.62E‐03	5.49E‐06	2.55E‐03
	200	5.35E‐06	5.79E‐03	1.41E‐05	5.87E‐03
	300	4.27E‐06	1.20E‐02	1.08E‐05	1.22E‐02
*Alnus*	100	−7.16E‐07	5.83E‐03	1.89E‐05	6.31E‐03
	200	−4.01E‐06	8.53E‐03	2.65E‐06	8.54E‐03
	300	−2.33E‐05	1.35E‐02	−5.84E‐05	1.16E‐02
*Fagus*	100	−1.63E‐06	1.25E‐03	−5.20E‐06	1.13E‐03
	200	−7.87E‐08	4.44E‐03	1.12E‐05	4.61E‐03
	300	7.42E‐06	1.53E‐02	3.80E‐05	1.62E‐02
*Larix*	100	7.11E‐07	2.13E‐03	−2.79E‐07	2.22E‐03
	200	1.47E‐06	1.03E‐02	5.87E‐06	1.06E‐02
	300	1.13E‐05	1.38E‐02	8.49E‐05	1.63E‐02
*Picea*	100	1.92E‐06	1.63E‐03	8.84E‐06	1.89E‐03
	200	1.49E‐05	1.64E‐02	8.46E‐06	1.73E‐02
	300	1.78E‐05	2.76E‐02	2.01E‐05	2.90E‐02
*Pinus*	100	−2.75E‐06	7.06E‐03	−6.46E‐06	6.96E‐03
	200	1.34E‐05	2.15E‐02	4.73E‐05	2.28E‐02
	300	3.71E‐05	2.41E‐02	1.82E‐04	2.87E‐02
*Quercus*	100	3.75E‐06	2.92E‐03	2.78E‐05	3.69E‐03
	200	1.31E‐05	8.85E‐03	9.35E‐05	1.12E‐02
	300	4.35E‐06	1.08E‐02	6.37E‐05	1.23E‐02

For individual tree species, the predictions showed divergent trends in volume loss under model a2 (Figure S6 and Table S6). Under RCP8.5, *k*_loss_‐values for *Quercus* decreased for all diameters (−0.01), which may be due higher variation in the dataset when including negative *k*‐values. This leads to deterioration in model performance (*R* ^2^: 0.58), with dbh being the most important variable for model building (*R* ^2^: 0.81). The thick bark of *Quercus* likely leads to an increase in dbh as it detaches during decomposition. *Pinus* was still the most sensitive tree species, exhibiting an increase in *k*_loss_‐values among all diameters (0.01–0.02) under severe climate change. In contrast to model b2, medium‐ to large‐diameter *Alnus* (0.01), and medium‐diameter *Picea* (0.01) showed a higher susceptibility. *Quercus* and *Abies* seemed to be less sensitive to climate change.

## 4 DISCUSSION

Elucidating the dynamics and drivers relating to snag volume and persistence is essential when aiming to develop strategies for including snags into forest management and nature conservation planning. Here, we investigated the drivers of snag volume, persistence and volume loss as part of the decomposition process at large spatial and temporal scales based on Austrian NFI data. This bridges an important existing gap, as large‐scale studies are so far limited in number and these are largely restricted to lying or overall deadwood volume (Meyer & Schmidt, 2011). Moreover, long‐term monitoring programmes such as NFI are crucial when aiming for reliable results on snag dynamics (Levrel et al., 2010).

### 4.1 Living volume and site variables affect standing deadwood volume

Snag volume was mainly driven by living volume stock, and elevation. Russell et al. (2012) modelled snag volume under different silvicultural treatments in the north‐eastern United States and support our findings on the influence of living volume stock on snag volume. In line with our expectations, elevation had a major influence on snag volume. Similar relationships have been reported by Bujoczek et al. (2021) for total deadwood volume in Polish forests and by Crecente‐Campo et al. (2016) for snag volume in Spanish forests. This seems to be primarily related to the limited accessibility of forests at high elevations and in steep terrain for timber harvesting (Merganiov et al., 2012; Parisi et al., 2018).

Contrary to our hypothesis (H I), forest type was of surprisingly low importance in our models, with snag volume ranging from 6.35 m^3^ ha^−1^ in deciduous hardwood forests to 11.32 m^3^ ha^−1^ in fir forests. One reason could be the legally prescribed salvage loggings to protect forest stands preventively or combatively from large‐scale insect infestation. In this context, the accumulation of snags and total deadwood is reduced independently from forest types. Contrary to our results, studies from Austrian as well as European unmanaged forest reserves and old‐growth forests have identified forest type as an important predictor of snag and total deadwood volume (Christensen et al., 2005; Nilsson et al., 2002; Oettel et al., 2020).

### 4.2 Intensive forest management reduces snag persistence

Snag persistence was lower under intensive than under extensive forest management. This is in accordance with our hypothesis (H II) and consistent with other studies showing negative effects of intensive forest management on snag residence time (Brunet & Isacsson, 2009; Hararuk et al., 2020). Our results support the general trend that high management intensities are negatively associated with the availability of deadwood, as in particular snags and large diameter deadwood is removed. For improving the quantity and quality of deadwood, passive restoration measures, such as the prolongation of rotation periods, the exclusion of snags from harvesting (Storch et al., 2018), and the targeted conservation of large‐dimensional deadwood and provision of advanced decomposition stages (Gossner et al., 2013), as well as active restoration measures, such as the artificial creation of standing deadwood by girdling, have been recommended (Bauhus et al., 2009; Ranius et al., 2005).

In line with our expectations (H II), persistence of snags differed significantly between ownership categories being noticeably higher in public forests compared to all other categories. This is likely due to the integrative nature conservation strategy implemented by the Austrian Federal forests (ÖBf) over the last decades, aiming at establishing an ecological landscape management (ÖBf, 2008). The strategy targets a minimum total deadwood volume of 25 m^3^ ha^−1^ in managed forests based on the retention of 7–10 (standing and lying) dead trees per ha with a dbh > 20 cm. In addition, retention of snags and trunks during harvesting activities is being enforced (ÖBf, 2017). Similar approaches are applied in other European countries (e.g. Ekbom et al., 2006; Gustafsson et al., 2012; Laarmann et al., 2009) and have been shown to be successful, that is, in Germany (Doerfler et al., 2017).

The range of snag persistence reported here (3–20 years) is lower than reported residence times in other studies, because we only examined the time from tree death to tree fall and did not include further decomposition. Comparison of results must therefore be considered carefully, as different definitions of residence time exist. Some authors define residence time as the time from tree death to final total decomposition (Přívětivý et al., 2016), or as the number of years after which a certain percentage of biomass remains (Přívětivý et al., 2018). Aakala (2010) reported snag residence times (until fall) of *Picea abies* for Northern European forests ranging between 12 and 27 years. Similarly, the probability of a snag remaining standing has been reported to drop below 50% 20–25 years after death for *Picea abies* in Finland (Mäkinen et al., 2006). These values are in the upper range of our reported snag persistence. However, since our observation period was limited to 28 years, longer observations were not possible. Future studies should separately investigate snag persistence and underlying processes until snag fall, as these have special microclimatic conditions that are particularly important for protecting and promoting an adapted community of organisms (Bouget et al., 2012).

### 4.3 Tree diameter and climatic factors influence volume loss

Our study is among few estimating volume loss of snags as part of the decomposition process (Fraver et al., 2013). We chose a linear model since the seven investigated tree genera showed linear snag volume loss behaviour over time, with average *k*_loss_‐values ranging from 0.010 year^−1^ (*Picea*) to 0.016 year^−1^ (*Pinus*). Our study on volume loss rates did not confirm the general trend towards higher decomposition rates for angiosperms than for gymnosperms, as reported by Kahl et al. (2017). However, the variance in volume loss rates among tree species was considerable, which seems to be the result of tree sizes and tree species‐specific wood properties (Bond‐Lamberty & Gower, 2008; Harmon et al., 1986; Ulyshen, 2016), as well as the varying sensitivity to climatic conditions (Herrmann & Bauhus, 2013; Pietsch et al., 2019).

There are different model variants for determining decomposition rates (*k*) describing decomposition over time as a linear, exponential, sigmoid or lag‐time process (Fraver et al., 2013; Harmon et al., 1986; Olsen, 1963; Pietsch et al., 2014) using dry mass (e.g. Fravolini et al., 2018; Herrmann et al., 2015) or density of wood as input variable (e.g. Kahl et al., 2017; Rock et al., 2008). The methodology applied for age estimation may further influence the results, making comparison among studies difficult (Petrillo et al., 2016). Volume loss, attributed to fragmentation as part of decomposition process (Harmon et al., 1986; Müller‐Using & Bartsch, 2009), has rarely been investigated separately (Fraver et al., 2013) or not considered at all, which may lead to underestimation of total decomposition rates (Herrmann & Prescott, 2008; Müller‐Using & Bartsch, 2009). Fragmentation further implies breakage into pieces (Fraver et al., 2013). Our model results, however, indicated that for a detailed investigation, broken and unbroken individuals or the process of volume loss until breakage should be studied separately. Therefore, care needs to be applied when comparing our rates in volume loss *k*_loss_ to decomposition or fragmentation rates from other studies.

In accordance with our hypothesis (H III), snag volume loss in Austrian forests was mainly driven by dbh, temperature, precipitation and relative humidity. The variable ranking clearly highlighted the importance of dbh for all tree genera, which corresponds to other studies, associating dbh with the time that snags remain standing on site (e.g. Merganiov et al., 2012; Zell et al., 2009). Relative humidity was of high importance for *Abies*, *Fagus* and *Quercus*, while temperature had a greater influence on *Alnus* and *Larix*. For *Pinus* and *Picea* temperature and relative humidity were of similar importance, indicating a combined effect. A similar temperature sensitivity has been reported for Swiss alpine (Hararuk et al., 2020), boreal (Shorohova & Kapitsa, 2014) and subtropical forests (Pietsch et al., 2019). The reason is that higher temperatures stimulate microbial activity (Suseela et al., 2012). Our results are supported by other studies reporting that high temperatures and sufficient humidity promote decomposer activity, leading to faster decomposition (Pietsch et al., 2014, 2019; Weedon et al., 2009).

### 4.4 Advancing climate change accelerates snag volume loss

Our predictions revealed constant *k*_loss_‐values for all tree genera and tree diameters under moderate climate change conditions using two different models (a2 and b2). Under severe climate change, *k*_loss_‐values increased, in line with our expectations (H IV), noticeably for *Pinus* and *Fagus*, which mainly occur at low elevations with high temperatures and low to medium precipitation amounts. By contrast, *Alnus, Quercus, Larix* and *Picea* were moderately susceptible and *Abies* was least sensitive to climate change. The latter occurred at sites with the highest precipitation amounts. Trends towards faster decomposition under warmer climate conditions at lower elevations and reduced ones under moist and cool climates have also been reported for the eastern and interior western United States (Garbarino et al., 2015; Russell et al., 2014) and generally for a Europe and America by means of a meta‐analysis (Chagnon et al., 2022). Considering that sites with high precipitation amounts are located at relatively high elevations, precipitation could represent a long snow cover, which may slow down decomposition processes (Chagnon et al., 2022; Gómez‐Brandón et al., 2020). In contrast, high temperatures and moderately moist conditions lead to increased saproxylic beetle, fungal and microbial activities (Müller et al., 2015; Van Der Wal et al., 2015; Yin, 1999). In general, the increase in *k*_loss_‐values was more pronounced towards the second half of the forecast period (2050–2100), when climate change has progressed further.

Climate is known to be important in estimating carbon fluxes (Woodall & Westfall, 2009), and deadwood decomposition is a major control of carbon storage in forests (Yatskov et al., 2003). Seibold et al. (2021) revealed that insects accelerate decomposition of deadwood directly and indirectly through interactions with micro‐organisms, resulting in a net effect of 29% of carbon flux from deadwood. Our findings on volume loss as part of the decomposition process and its behaviour under changing climatic conditions provide valuable insights on the role of forests for carbon fluxes under climate change. More rapid volume loss, as predicted under severe climate change conditions, implies carbon emissions from forests (Chagnon et al., 2022; Kahl et al., 2017; Pietsch et al., 2014). The most susceptible tree species, such as *Pinus and Fagus*, are especially relevant in this context, as a more rapid decomposition will reduce temporal availability of species‐specific deadwood. Considering the decomposition rates for lying deadwood reported by previous studies (e.g. Herrmann et al., 2015; Kahl et al., 2017), a similar or even more pronounced trend in decomposition behaviour under climate change scenarios can be assumed for lying deadwood due to soil contact and a correspondingly higher wood moisture content (Harmon et al., 2000). The decomposition of standing and lying deadwood should therefore be investigated separately—including information on mineralization (leaching and respiration) and fragmentation behaviour as well as organismic activity in different stages of decomposition—to draw more precise conclusions.

More rapid decomposition of deadwood under climate change conditions has implications for forest biodiversity as well. Organisms, such as fungi and Coleoptera are important agents of wood decomposition (Seibold et al., 2021). Van Der Wal et al. (2015), Kahl et al. (2017) and Seibold et al. (2021) reported accelerated decomposition with increasing diversity of deadwood colonizers. Our prediction of more rapid volume loss will lead to a shorter availability of snags as habitats for species with long development cycles (Chagnon et al., 2022), many of which are considered rare or threatened. The continuity of deadwood supply in various stages of decomposition is one of the most crucial factors for the survival of many threatened bryophytes, lichens, fungi, insects and birds (e.g. Humphrey et al., 2005; Jonsell et al., 1998). Accordingly, there is an urgent need to further examine the consequences of climate change on snag decomposition, persistence and the resulting potential consequences for deadwood‐dependent species. In addition, the response of species life cycles under climate change conditions should be investigated as well. Linking information on trends in snag decomposition with trends in the development of dependent species will improve our understanding of the consequences of climate change and allow conclusions to be drawn with regard to adapting deadwood management.

To conclude, for biodiversity‐oriented forest management in the face of climate change, the enrichment of snags is essential. We recommend particular emphasis on the active promotion of the most climate‐sensitive genera, such as *Pinus* and *Fagus*, and excluding them from management where reasonable and appropriate.

## AUTHOR CONTRIBUTIONS

Janine Oettel, Katharina Lapin, Martin M. Gossner and Franz Essl substantially contributed to the conception and design of the present research. Thomas Gschwantner and Georg Kindermann were responsible for data acquisition. Janine Oettel, Anita Zolles, Thomas Gschwantner and Karl‐Manfred Schweinzer processed the data and performed the analysis. Franz Essl and Martin M. Gossner supervised Janine Oettel in interpreting the data. Janine Oettel led the writing of the manuscript with substantial input from all co‐authors. All authors gave their final approval for the publication of the manuscript.

## CONFLICT OF INTEREST

The authors declare that they have no known competing financial interests or personal relationships that could have appeared to influence the work reported in this paper.

## Supporting information


**Figure S1.** Snag dbh (diameter at breast height [mm] and height [dm]) measurement values of the seventh Austrian NFI (National Forest Inventory) period (*n* = 1417). Light blue points indicate broken individuals (*n* = 601, 42.4%).
**Figure S2.** Linear relationship (light blue line) between initial snag volume [m3] measured after tree death (initial volume) and the volume measured before snag fall (final volume) for the tree genera Abies (R^2^:0.99), Alnus (R^2^:0.95), Fagus (R^2^:0.98), Larix (R^2^:0.98), Picea (R^2^:0.94), Pinus (R^2^:0.95), Quercus (R^2^:0.99).The linear relationship is visualized for all tree genera with a 1:1 relationship (grey dashed line).
**Figure S3.** Density estimates of the volume loss rate k_loss_ [year^−1^] per tree genus. The estimate was performed with a Gaussian kernel and a bandwidth of 0.01.
**Figure S4a–f.** Trend and magnitude between snag volume [m^3^ha^−1^] and the influencing predictors based on observations for (a) living volume stock [m^3^ha‐^1^], (b) elevation [m], (c) NFI survey period (NFI3‐NFI7) using linear regression plots and (d) forest ownership, (e) forest type and (f) forest management intensity using boxplots. Median values, 25% and 75% percentiles (boxes), min‐max values (whiskers) and outliers are presented. A description of variables is presented in Table S1.
**Figure S5a.** Comparison of observed with modelled values for deadwood volume loss rate k_loss_ based on model a2 (negative and positive k‐values, excluding broken trees) for seven tree genera.
**Figure S5b.** Comparison of observed with modelled values for deadwood volume loss rate k_loss_ based on model b2 (only positive k‐values, excluding broken trees) for seven tree genera.
**Figure S6.** Prediction of snag volume loss rate k_loss_ [year^−1^] using model a2 as baseline condition under climate change scenarios RCP2.6 and RCP8.5 for the seven investigated tree genera. Subplots refer to different tree diameters at breast height (dbh: 100, 200, 300 mm). To enable a comparison of volume loss among the investigated tree genera, the k_loss_‐values are expressed as deviation from the respective median. The slopes and averages of the individual tree genera per diameter at breast height (dbh) are provided in Table S6.
**Table S1.** Tree‐, stand‐, site‐ and management‐related variables as assessed by the Austrian National Forest Inventory (NFI) as well as climate‐related variables.
**Table S2.** Ecoregions in Austria with largely uniform environmental factors described as elevational and climatic ranges adapted from Kilian, Müller and Starlinger (1994).
**Table S3.** Formed classes of snag volume per hectare with almost equal number of plots per class. Bold number indicates the number of cases used for the training set of the volume model.
**Table S4.** Volume loss model variants a1 to b2 for the main occurring coniferous (Picea) and deciduous (Fagus) tree species. The table shows variable importance and overall model performance expressed as R^2^.
**Table S5a.** Correlations between volume loss constant k_loss_ [year^−1^] and the influencing predictors temperature [°C], precipitation [mm], relative humidity [%], elevation [m] and diameter at breast height (dbh) [mm] for the seven investigated tree genera based on observations (dataset 3 for model a2). A description of variables is presented in Table S1.
**Table S5b.** Correlations between volume loss constant k_loss_ [year^−1^] and the influencing predictors temperature [°C], precipitation [mm], relative humidity [%], elevation [m] and diameter at breast height (dbh) [mm] for the seven investigated tree genera based on observations (dataset 3 for **model b2**). A description of variables is presented in Table S1.
**Table S6.** Slopes and averages of k_loss_‐values using **model a2** as baseline condition for the seven investigated tree genera per diameter at breast height (dbh: 100, 200, 300 mm) under different climate change scenarios.

## Data Availability

National Forest Inventory data are not publicly available. The data that support the findings of this study are available upon reasonable request and permission from the Austrian National Forest Inventory (NFI). Information and further description is available online via https://waldinventur.at/#/ENG.
