# Supplementary material for: Dynamics of standing deadwood in Austrian forests under varying forest management and climatic conditions
Source: J Appl Ecol. 2023 Jan 24;60(4):696–713. doi: 10.1111/1365-2664.14359 (PMC10947403; doi:10.1111/1365-2664.14359)
Supplement: Supplementary file 1 — Figure S1. Snag dbh (diameter at breast height [mm] and height [dm]) measurement values of the seventh Austrian NFI (National Forest Inventory) period (n = 1417). Light blue points indicate broken individuals (n = 601, 42.4%). Figure S2. Linear relationship (light blue line) between initial snag volume [m3] measured after tree death (initial volume) and the volume measured before snag fall (final volume) for the tree genera Abies (R2:0.99), Alnus (R2:0.95), Fagus (R2:0.98), Larix (R2:0.98), Picea (R2:0.94), Pinus (R2:0.95), Quercus (R2:0.99).The linear relationship is visualized for all tree genera with a 1:1 relationship (grey dashed line). Figure S3. Density estimates of the volume loss rate kloss [year−1] per tree genus. The estimate was performed with a Gaussian kernel and a bandwidth of 0.01. Figure S4a–f. Trend and magnitude between snag volume [m3ha−1] and the influencing predictors based on observations for (a) living volume stock [m3ha‐1], (b) elevation [m], (c) NFI survey period (NFI3‐NFI7) using linear regression plots and (d) forest ownership, (e) forest type and (f) forest management intensity using boxplots. Median values, 25% and 75% percentiles (boxes), min‐max values (whiskers) and outliers are presented. A description of variables is presented in Table S1. Figure S5a. Comparison of observed with modelled values for deadwood volume loss rate kloss based on model a2 (negative and positive k‐values, excluding broken trees) for seven tree genera. Figure S5b. Comparison of observed with modelled values for deadwood volume loss rate kloss based on model b2 (only positive k‐values, excluding broken trees) for seven tree genera. Figure S6. Prediction of snag volume loss rate kloss [year−1] using model a2 as baseline condition under climate change scenarios RCP2.6 and RCP8.5 for the seven investigated tree genera. Subplots refer to different tree diameters at breast height (dbh: 100, 200, 300 mm). To enable a comparison of volume loss among the investigated [file JPE-60-696-s001.zip › JPE_14359_Supporting Information.docx]

Supporting Information

**Dynamics of standing deadwood in Austrian forests under varying forest management and climatic conditions**

**Authors**

Janine Oettel^1^*, Anita Zolles^1^, Thomas Gschwantner^1^, Katharina Lapin^1^, Georg Kindermann^1^, Karl-Manfred Schweinzer^1^, Martin M. Gossner^2,3^ ª, Franz Essl^4^ ª

^1^ Austrian Federal Research Centre for Forests, Natural Hazards and Landscape (BFW), Seckendorff-Gudent-Weg 8, 1131 Vienna, Austria

^2^ Forest Entomology, Swiss Federal Research Institute of Forest, Snow and Landscape Research (WSL), Zürcherstrasse 111, 8903 Birmensdorf, Switzerland

^3^ ETH Zurich, Department of Environmental Systems Science, Institute of Terrestrial Ecosystems, 8092 Zurich, Switzerland

^4^ BioInvasions, Global Change, Macroecology-Group, Department of Botany and Biodiversity Research, University Vienna, Rennweg 14, 1030 Vienna, Austria

**Table of Contents**

**Table S1.** Tree-, stand-, site- and management-related variables as assessed by the Austrian National Forest Inventory (NFI) as well as climate-related variables.

**Table S2**. Ecoregions in Austria with largely uniform environmental factors described as elevational and climatic ranges adapted from Kilian, Müller and Starlinger (1994).

**Table S3.** Formed classes of snag volume per hectare with almost equal number of plots per class. Bold number indicates the number of cases used for the training set of the volume model.

**Table S4.** Volume loss model variants a1 to b2 for the main occurring coniferous (Picea) and deciduous (Fagus) tree species. The table shows variable importance and overall model performance expressed as R^2^.

**Table S5a.** Correlations between volume loss constant *k_loss_* [year^-1^] and the influencing predictors temperature [°C], precipitation [mm], relative humidity [%], elevation [m] and diameter at breast height (dbh) [mm] for the seven investigated tree genera based on observations (data set 3 for model a2). A description of variables is presented in Table S1.

**Table S5b.** Correlations between volume loss constant *k_loss_* [year^-1^] and the influencing predictors temperature [°C], precipitation [mm], relative humidity [%], elevation [m] and diameter at breast height (dbh) [mm] for the seven investigated tree genera based on observations (data set 3 for **model b2**). A description of variables is presented in Table S1.

**Table S6.** Slopes and averages of k_loss_ -values using **model a2** as baseline condition for the seven investigated tree genera per diameter at breast height (dbh: 100, 200, 300 mm) under different climate change scenarios.

**Figure S1.** Snag dbh (diameter at breast height [mm] and height [dm] measurement values of the seventh Austrian NFI (National Forest Inventory) period (n=1417). Light blue points indicate broken individuals (n=601, 42.4%).

**Figure S2.** Linear relationship (light blue line) between initial snag volume [m3] measured after tree death (initial volume) and the volume measured before snag fall (final volume) for the tree genera *Abies* (R^2^:0.99), *Alnus* (R^2^:0.95), *Fagus* (R^2^:0.98, *Larix* (R^2^:0.98), *Picea* (R^2^:0.94), *Pinus* (R^2^:0.95), *Quercus* (R^2^:0.99).The linear relationship is visualized for all tree genera with a 1:1 relationship (grey dashed line).

**Figure S3.** Density estimates of the volume loss rate *k_loss_* [year^-1^] per tree genus. The estimate was performed with a Gaussian kernel and a bandwidth of 0.01.

**Figure S4a-f.** Trend and magnitude between snag volume [m^3^ha^-1^] and the influencing predictors based on observations for (a) living volume stock [m^3^ha-^1^], (b) elevation [m], (c) NFI survey period (NFI3-NFI7) using linear regression plots and (d) forest ownership, (e) forest type and (f) forest management intensity using boxplots. Median values, 25% and 75% percentiles (boxes), min-max values (whiskers) and outliers are presented. A description of variables is presented in Table S1.

**Figure S5a.** Comparison of observed with modelled values for deadwood volume loss rate k_loss_ based on model a2 (negative and positive k -values, excluding broken trees) for seven tree genera.

**Figure S5b.** Comparison of observed with modelled values for deadwood volume loss rate k_loss_ based on model b2 (only positive k-values, excluding broken trees) for seven tree genera.

**Figure S6.** Prediction of snag volume loss rate k_loss_ [year^-1^] using **model a2** as baseline condition under climate change scenarios RCP2.6 and RCP8.5 for the seven investigated tree genera. Subplots refer to different tree diameters at breast height (dbh: 100, 200, 300 mm). To enable a comparison of volume loss among the investigated tree genera, the k_loss_-values are expressed as deviation from the respective median. The slopes and averages of the individual tree genera per diameter at breast height (dbh) are provided in Table S6.

**Table S1.** Tree-, stand-, site- and management-related variables as assessed by the Austrian National Forest Inventory (NFI) as well as climate-related variables and used for the analysis

| **Category** | **Variable** | **Acronym** | **Unit** | **Classification** | **Range/Categories** |
| --- | --- | --- | --- | --- | --- |
| Tree | Tree species | species | - | Categorical | *Abies* spp. |
|  |  |  |  |  | *Acer* spp. |
|  |  |  |  |  | *Alnus* spp. |
|  |  |  |  |  | *Betula* spp. |
|  |  |  |  |  | *Carpinus* spp. |
|  |  |  |  |  | *Castanea* spp. |
|  |  |  |  |  | *Fagus* spp. |
|  |  |  |  |  | *Fraxinus* spp. |
|  |  |  |  |  | *Larix* spp. |
|  |  |  |  |  | *Picea* spp. |
|  |  |  |  |  | *Pinus* spp. |
|  |  |  |  |  | *Populus* spp. |
|  |  |  |  |  | *Quercus* spp. |
|  |  |  |  |  | *Robinia* spp. |
|  |  |  |  |  | *Sorbus* spp. |
|  |  |  |  |  | *Ulmus* spp. |
|  | Diameter at breast height | dbh | mm | Continuous | 50–2,015 |
|  | Residence time |  | years | Continuous | 3–20 |
| Stand | Standing deadwood volume | Vsnag | m^3^ha^-1^ | Continuous | 0–508.3 |
|  | Living volume | Vliving | m^3^ha^-1^ | Continuous | 0–1662.2 |
|  | Crown cover | crown_1 | - | Categorical | gaps, clustered (space for more than one crown) |
|  |  | crown_2 |  |  | open (space for one more crown) |
|  |  | crown_3 |  |  | light (space for less than one crown) |
|  |  | crown_4 |  |  | closed (crowns touch each other) |
|  |  | crown_5 |  |  | dense (crowns grow into each other) |
|  | Forest type | foresttype_1 | - | Categorical | spruce forest |
|  |  | foresttype_2 |  |  | fir forest |
|  |  | foresttype_3 |  |  | pine forest |
|  |  | foresttype_4 |  |  | larch forest |
|  |  | foresttype_5 |  |  | swiss stone pine forest |
|  |  | foresttype_6 |  |  | coniferous non-native forest |
|  |  | foresttype_8 |  |  | beech forest |
|  |  | foresttype_9 |  |  | oak forest |
|  |  | foresttype_10 |  |  | deciduous hardwood forest |
|  |  | foresttype_11 |  |  | deciduous softwood forest |
|  |  | foresttype_12 |  |  | deciduous non-native forest |

**Table S1.** continued …

| **Category** | **Variable** | **Acronym** | **Unit** | **Classification** | **Range/Categories** |
| --- | --- | --- | --- | --- | --- |
| Site | Eco-region | eco_region_1.1 | - | Categorical classification | inner region of the Alps - continental core zone |
|  |  | eco_region_1.2 |  | of Kilian et al. (1994) | inner region of the Alps - subcontinental west part |
|  |  | eco_region_1.3 |  |  | inner region of the Alps - subcontinental east part |
|  |  | eco_region_2.1 |  |  | northern transitional region of the Alps - west part |
|  |  | eco_region_2.2 |  |  | northern transitional region of the Alps - east part |
|  |  | eco_region_3.1 |  |  | eastern transitional region of the Alps - north part |
|  |  | eco_region_3.2 |  |  | eastern transitional region of the Alps - south part |
|  |  | eco_region_3.3 |  |  | southern transitional region of the Alps |
|  |  | eco_region_4.1 |  |  | northern peripheral region of the Alps - west part |
|  |  | eco_region_4.2 |  |  | northern peripheral region of the Alps - east part |
|  |  | eco_region_5.1 |  |  | eastern peripheral region of the Alps - Thermenalpen |
|  |  | eco_region_5.2 |  |  | eastern peripheral region of the Alps - Buckige Welt |
|  |  | eco_region_5.3 |  |  | eastern peripheral region of the Alps - east and central Styrian part |
|  |  | eco_region_5.4 |  |  | eastern peripheral region of the Alps - west Styrian part |
|  |  | eco_region_6.1 |  |  | southern peripheral region of the Alps - south margin part |
|  |  | eco_region_6.2 |  |  | southern peripheral region of the Alps - Klagenfurt Basin |
|  |  | eco_region_7.1 |  |  | northern alpine upland - west part |
|  |  | eco_region_7.2 |  |  | northern alpine upland - east part |
|  |  | eco_region_8.1 |  |  | eastern lowlands - Pannonian lowlands and hills |
|  |  | eco_region_8.2 |  |  | eastern lowlands - subillyrian hills and terraces |
|  |  | eco_region_9.1 |  |  | Bohemian Massif - Mühlviertel |
|  |  | eco_region_9.2 |  |  | Bohemian Massif - Waldviertel |
|  | Elevation | height_asl | m asl | Continuous | 116–2015 |

**Table S1.** continued …

| **Category** | **Variable** | **Acronym** | **Unit** | **Classification** | **Range/Categories** |
| --- | --- | --- | --- | --- | --- |
| Site | Slope | slope_0 | % | Categorical | 0–5 |
|  |  | slope_1 |  | (grouped % values) | 6–10 |
|  |  | slope_2 |  |  | 11–20 |
|  |  | slope_3 |  |  | 21–30 |
|  |  | slope_4 |  |  | 31–40 |
|  |  | slope_5 |  |  | 41–50 |
|  |  | slope_6 |  |  | 51–60 |
|  |  | slope_7 |  |  | 61–70 |
|  |  | slope_8 |  |  | 71–80 |
|  |  | slope_9 |  |  | 81–90 |
|  |  | slope_10 |  |  | 91–100 |
|  |  | slope_11 |  |  | 101–110 |
|  |  | slope_12 |  |  | >110 |
|  | Aspect | aspect_0 | gon | Categorical | 0 |
|  |  | aspect_1 |  | (grouped gon values) | 1–25; 376–399 |
|  |  | aspect_2 |  |  | 26–75 |
|  |  | aspect_3 |  |  | 76–125 |
|  |  | aspect_4 |  |  | 126–175 |
|  |  | aspect_5 |  |  | 176–225 |
|  |  | aspect_6 |  |  | 226–275 |
|  |  | aspect_7 |  |  | 276–325 |
|  |  | aspect_8 |  |  | 326–375 |
| Management | Fall reason | natural | - | Categorical | natural disintegration |
|  |  | manage | - |  | management induced disintegration (felling) |
|  | Forest ownership | ownership_1 | - | Categorical | small scale forest (≤ 200 ha) |
|  |  | ownership_3 |  |  | public ÖBf (federal owned forest) |
|  |  | ownership_4 |  |  | medium scale private forest (> 200 ha and ≤ 1,000 ha) |
|  |  | ownership_5 |  |  | large scale private forest (> 1,000 ha) |
|  |  | ownership_6 |  |  | public (state, municipal) owned forest (> 200 ha) |
|  | Forest management intensity | intensity_1 | - | Categorical | intensive |
|  |  | intensity_2 |  |  | extensive |
| Climate | Temperature |  | °C | Continuous | -24.7–29.7 |
|  | Precipitation |  | mm | Continuous | 0.0–502.0 |
|  | Relative Humidity |  | % | Continuous | 29–99 |

**Table S1.** continued …

| **Category** | **Variable** | **Acronym** | **Unit** | **Classification** | **Range/Categories** |
| --- | --- | --- | --- | --- | --- |
| NFI | NFI survey period | Survey period | - | Categorical | NFI 3 (1981–1985) |
|  |  |  |  |  | NFI 4 (1986–1990) |
|  |  |  |  |  | NFI 5 (1992–1996) |
|  |  |  |  |  | NFI 6 (2000–2002) |
|  |  |  |  |  | NFI 7 (2007–2009) |

**Table S2.** Ecoregions in Austria with largely uniform environmental factors described as elevational and climatic ranges adapted from Kilian, Müller and Starlinger (1994).

| **Major ecoregion** | **Ecoregion** | **Description** | **Range in elevation (m)** | **Description of climate** | **Range in annual precipitation sum (mm)** |
| --- | --- | --- | --- | --- | --- |
| ecoregion 1 | eco_region_1.1 | inner region of the Alps - continental core zone | 650-3772 | continental inner alpine climate; driest area of the Austrian Alpine region | 600-1000 |
|  | eco_region_1.2 | inner region of the Alps - subcontinental west part | 560-3797 | continental inner alpine climate; higher precipitation than continental core zone (eco_region_1.1) | 800-1250 |
|  | eco_region_1.3 | inner region of the Alps - subcontinental east part | 750-3797 | moderate inner alpine climate; continental climate remains only in sheltered valleys (Lungau, Millnitz, Murtal) | 800-1200 |
| ecoregion 2 | eco_region_2.1 | northern transitional region of the Alps - west part | 500-3312 | transitional climate from sub-continental dry inner Alps to the cool-humid peripheral Alps | 1000-1900 |
|  | eco_region_2.2 | northern transitional region of the Alps - east part | 640-2995 | transitional climate with inversion in some valleys (Enns valey, Zell am See); less pronounced than in northern transitional region (eco_region_2.1) | 1000-1500 |
| ecoregion 3 | eco_region_3.1 | eastern transitional region of the Alps - north part | 490-2448 | a pronounced precipitation maximum in july; less precipitation than the northern transitional Alps | 750-1500 |
|  | eco_region_3.2 | eastern transitional region of the Alps - south part | 460-2448 | milder climate than in the northern part of the eastern transitional Alps (eco_egion_3.1); basin locations (Murtal, Friesach) are of inner alpine character | 800-1500 |
|  | eco_region_3.3 | southern transitional region of the Alps | 505-2965 | higher precipitation than in inner alps (ecoregion 1) due to southern exposure; higher precipitation than in eastern transitional Alps (eco_region_3.2) | 900-1200 |
| ecoregion 4 | eco_region_4.1 | northern peripheral region of the Alps - west part | 395-2995 | cool humid climate with considerable local variation in precipitation totals | 1100-2500 |
|  | eco_region_4.2 | northern peripheral region of the Alps - east part | 312-2369 | cool humid climate; lower precipitation than in the western part (eco_region_4.1); high winter precipitation in the mountain areas, decrease towards the east | 1000-1900 |
|  |  |  |  |  |  |

| **Table S2.** continued … | | | | | |
| --- | --- | --- | --- | --- | --- |
| **Major ecoregion** | **Ecoregion** | **Description** | **Range in elevation (m)** | **Description of climate** | **Range in annual precipitation sum (mm)** |
| ecoregion 5 | eco_region_5.1 | eastern peripheral region of the Alps - Thermenalpen | 170-2076 | transitional climate from moist peripheral region to pannonian-influenced eastern peripheral region with a pronounced precpitation gradient in west-east direction | 700-1250 |
|  | eco_region_5.2 | eastern peripheral region of the Alps - Buckige Welt | 320-1743 | cool and less illyrian climate than eco_region_5.3 and eco_region_5.4 | 700-1250 |
| ecoregion 5  ecoregion 6 | eco_region_5.3 | eastern peripheral region of the Alps - east and central Styrian part | 292-1988 | increasing sub-illyrian influence from northeast to southwest; high humidity and thunderstorm frequency; increasing precipitation from Burgenland to southwest | 700-1100 |
|  | eco_region_5.4 | eastern peripheral region of the Alps - west Styrian part | 314-2140 | sub-illyrian influence, especiall in southeast part; high humidity; higher precipitation than in east and central part (eco_region_5.3) | 900-1500 |
|  | eco_region_6.1 | southern peripheral region of the Alps - south margin part | 348-2780 | distinct illyrian climate influence; high humidity and precipitation with a sub-mediterranean maximum in autumn | 1200-2000 |
|  | eco_region_6.2 | southern peripheral region of the Alps - Klagenfurt Basin | 348-1069 | continentally influenced climate, but higher precipitation due to peripheral region | 700-1500 |
| ecoregion 7 | eco_region_7.1 | northern alpine upland - west part | 313-801 | oceanic climate with high summer precipitation | 800-1500 |
|  | eco_region_7.2 | northern alpine upland - east part | 228-553 | oceanic climate; less precipitation and milder than the west part of the nothern alpine upland (eco_region_7.1) | 600-1000 |
| ecoregion 8 | eco_region_8.1 | eastern lowlands - Pannonian lowlands and hills | 121-491 | pannonic-subcontinental climate; dry and warm; mild winter; dry periods in summer possible with frequent dry SE winds | 450-800 |
|  | eco_region_8.2 | eastern lowlands - subillyrian hills and terraces | 205-670 | sub-illyrian influence, similar temperature conditions and noticeably higher precipition compared to the Pannonian lowlands (eco_region_8.1) | 700-1000 |
|  |  |  |  |  |  |
|  |  |  |  |  |  |

| **Table S2.** continued … | | | | | |
| --- | --- | --- | --- | --- | --- |
| **Major ecoregion** | **Ecoregion** | **Description** | **Range in elevation (m)** | **Description of climate** | **Range in annual precipitation sum (mm)** |
| ecoregion 9 | eco_region_9.1 | Bohemian Massif - Mühlviertel | 222-1378 | cool climate of slightly boreal character, precipitation higher than in Waldviertel (eco_region_9.2) | 700-1100 |
|  | eco_region_9.2 | Bohemian Massif - Waldviertel | 205-1060 | cool and boreal climate; less precipitation than in Mühlviertel (eco_region_9.1) | 500-1000 |

**Table S3.** Formed classes of snag volume per hectare with almost equal number of plots per class. Bold number indicates the number of cases used for the training set of the volume model.

| **Category** | **Min deadwood volume (m3)** | **Max deadwood volume (m3)** | **n plots (observed during five periods of NFI)** |
| --- | --- | --- | --- |
| 0 | 0.00 | 0.00 | 5999 |
| 1 | 0.01 | 10.00 | 139 |
| 2 | 10.01 | 20.00 | 147 |
| 3 | 20.01 | 30.00 | 211 |
| 4 | 30.01 | 40.00 | 201 |
| 5 | 40.01 | 50.00 | 127 |
| 6 | 50.01 | 70.00 | **122** |
| 7 | 70.01 | 508.30 | 124 |

**Table S4.** Volume loss model variants a1 to b2 for the main occurring coniferous (Picea) and deciduous (Fagus) tree species. The table shows variable importance and overall model performance expressed as R^2^.

| Variable | ***Fagus*** | | | | ***Picea*** | | | |
| --- | --- | --- | --- | --- | --- | --- | --- | --- |
|  | model a1 | model a2 | model b1 | model b2 | model a1 | model a2 | model b1 | model b2 |
| elevation | 0.13 | 0.13 | 0.12 | 0.12 | 0.11 | 0.10 | 0.12 | 0.08 |
| dbh | 0.60 | 0.54 | 0.42 | 0.54 | 0.57 | 0.64 | 0.55 | 0.71 |
| temperature | 0.05 | 0.09 | 0.12 | 0.08 | 0.13 | 0.08 | 0.11 | 0.07 |
| precipitation | 0.02 | 0.11 | 0.06 | 0.03 | 0.07 | 0.08 | 0.11 | 0.07 |
| relative humidity | 0.20 | 0.14 | 0.28 | 0.23 | 0.13 | 0.10 | 0.12 | 0.06 |
| R^2^ | 0.64 | 0.79 | 0.98 | 0.99 | 0.95 | 0.87 | 0.37 | 0.75 |

**Table S5a.** Correlations between volume loss constant k_loss_ [year^-1^] and the influencing predictors temperature [°C], precipitation [mm], relative humidity [%], elevation [m] and diameter at breast height (dbh) [mm] for the seven investigated tree genera based on observations (data set 3 for model a2). A description of variables is presented in Table S1.

| **tree genus** | **dbh** | **elevation** | **temperature** | **precipitation** | **relative humidity** |
| --- | --- | --- | --- | --- | --- |
| *Abies* | 0.052 | 0.093 | -0.150 | 0.037 | 0.000 |
| *Alnus* | -0.066 | 0.081 | -0.055 | 0.043 | 0.147 |
| *Fagus* | 0.136 | 0.094 | -0.093 | 0.130 | 0.168 |
| *Larix* | -0.257 | 0.011 | 0.026 | 0.020 | -0.012 |
| *Picea* | 0.163 | 0.022 | -0.021 | 0.039 | 0.033 |
| *Pinus* | 0.147 | 0.018 | 0.080 | 0.062 | 0.101 |
| *Quercus* | -0.590 | 0.119 | -0.004 | 0.079 | 0.104 |
| **all** | 0.123 | 0.022 | -0.018 | 0.042 | 0.039 |

**Table S5b.** Correlations between volume loss constant k_loss_ [year^-1^] and the influencing predictors temperature [°C], precipitation [mm], relative humidity [%], elevation [m] and diameter at breast height (dbh) [mm] for the seven investigated tree genera based on observations (data set 3 for model b2). A description of variables is presented in Table S1.

| **tree genus** | **dbh** | **elevation** | **temperature** | **precipitation** | **relative humidity** |
| --- | --- | --- | --- | --- | --- |
| *Abies* | 0.580 | 0.381 | -0.401 | 0.297 | 0.195 |
| *Alnus* | 0.451 | 0.017 | -0.034 | 0.073 | 0.219 |
| *Fagus* | 0.535 | 0.207 | -0.139 | 0.168 | 0.253 |
| *Larix* | 0.498 | 0.133 | -0.088 | -0.060 | -0.125 |
| *Picea* | 0.417 | 0.059 | -0.067 | 0.074 | 0.037 |
| *Pinus* | 0.605 | -0.114 | 0.132 | -0.032 | -0.026 |
| *Quercus* | 0.651 | 0.145 | 0.022 | 0.278 | 0.023 |
| **all** | **0.435** | **0.042** | **-0.049** | **0.068** | **0.039** |

**Table S6.** Slopes and averages of k_loss_-values using model a2 as baseline condition for the seven investigated tree genera per diameter at breast height (dbh: 100, 200, 300 mm) under different climate change scenarios until the end of the 21^st^ century.

| **Tree genera** | **dbh** | **RCP2.6** | | **RCP8.5** | |
| --- | --- | --- | --- | --- | --- |
|  |  | slope | average | slope | average |
| *Abies* | 100 | -2.38E-05 | -1.72E-02 | -8.43E-06 | -1.85E-02 |
|  | 200 | -2.40E-05 | -1.65E-02 | -7.90E-06 | -1.79E-02 |
|  | 300 | -3.11E-05 | -1.48E-02 | -2.46E-05 | -1.64E-02 |
| *Alnus* | 100 | 8.52E-06 | 1.92E-04 | 4.73E-05 | 1.29E-03 |
|  | 200 | 1.03E-05 | -4.69E-04 | 6.52E-05 | 8.29E-04 |
|  | 300 | -3.52E-06 | -1.59E-03 | 1.80E-05 | -1.30E-03 |
| *Fagus* | 100 | 4.21E-06 | -1.29E-04 | 4.58E-05 | 9.76E-04 |
|  | 200 | 2.69E-06 | -6.10E-03 | 5.35E-05 | -5.00E-03 |
|  | 300 | 3.97E-06 | -1.77E-02 | 6.68E-05 | -1.65E-02 |
| *Larix* | 100 | 7.96E-06 | -4.41E-04 | 5.90E-05 | 1.07E-03 |
|  | 200 | 7.45E-06 | -4.67E-03 | 6.99E-05 | -3.34E-03 |
|  | 300 | -8.70E-07 | -4.60E-03 | 4.12E-05 | -4.09E-03 |
| *Picea* | 100 | 2.49E-06 | -2.00E-04 | 2.41E-05 | 4.47E-04 |
|  | 200 | 1.35E-05 | -6.86E-04 | 8.52E-05 | 1.39E-03 |
|  | 300 | -5.71E-06 | -1.30E-03 | -2.45E-05 | -2.62E-03 |
| *Pinus* | 100 | 1.56E-05 | 2.22E-03 | 7.78E-05 | 4.24E-03 |
|  | 200 | 5.75E-05 | 1.42E-04 | 2.55E-04 | 7.14E-03 |
|  | 300 | 4.95E-05 | 2.34E-03 | 2.38E-04 | 8.92E-03 |
| *Quercus* | 100 | -1.55E-06 | -3.76E-04 | -5.94E-05 | -2.12E-03 |
|  | 200 | -2.67E-06 | -2.46E-04 | -8.26E-05 | -2.70E-03 |
|  | 300 | -5.02E-06 | 6.73E-05 | -9.20E-05 | -2.62E-03 |


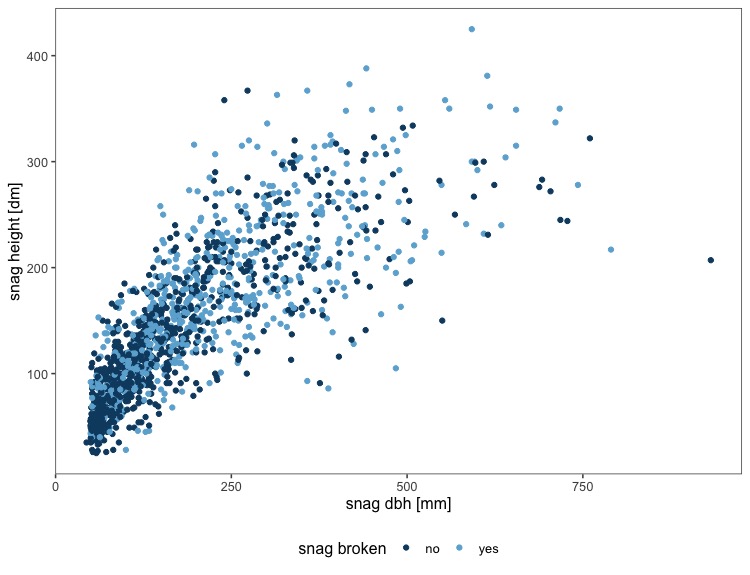


**Figure S1.** Snag dbh (diameter at breast height [mm] and height [dm] measurement values of the seventh Austrian NFI (National Forest Inventory) period (n=1417). Light blue points indicate broken individuals (n=601, 42.4%).


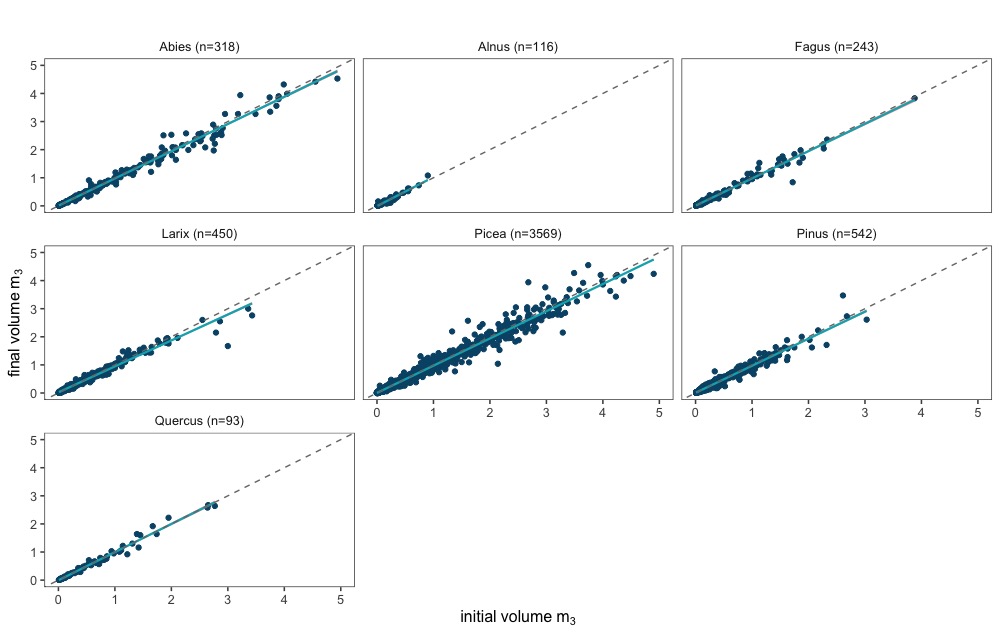
 **Figure S2.** Linear relationship (light blue line) between initial snag volume [m3] measured after tree death (initial volume) and the volume measured before snag fall (final volume) for the tree genera *Abies* (R^2^:0.99), *Alnus* (R^2^:0.95), *Fagus* (R^2^:0.98, *Larix* (R^2^:0.98), *Picea* (R^2^:0.94), *Pinus* (R^2^:0.95), *Quercus* (R^2^:0.99).The linear relationship is visualized for all tree genera with a 1:1 relationship (grey dashed line).


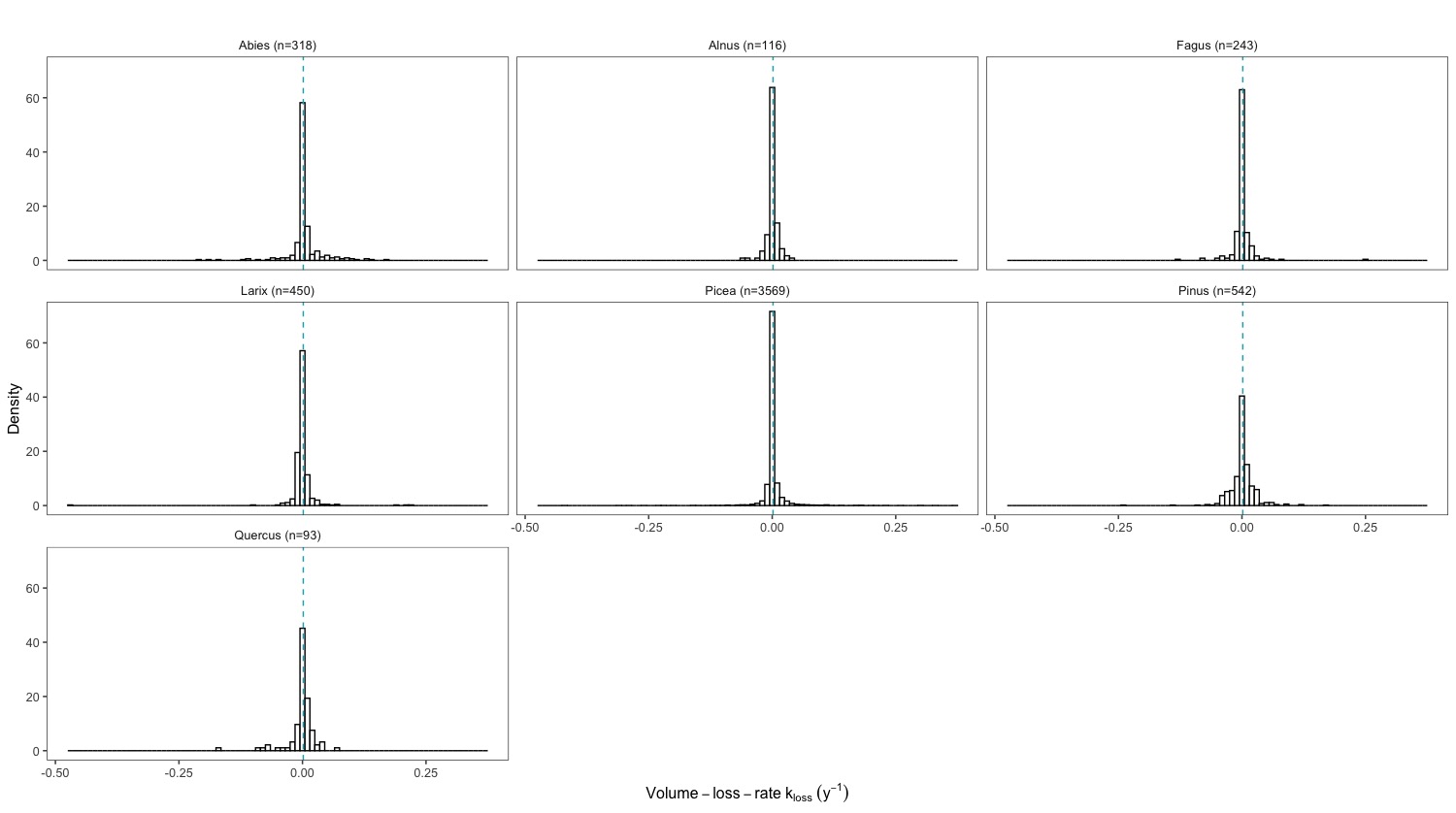


**Figure S3.** Density estimates of the volume loss rate *k_loss_* [year^-1^] per tree genus. The estimate was performed with a Gaussian kernel and a bandwidth of 0.01.


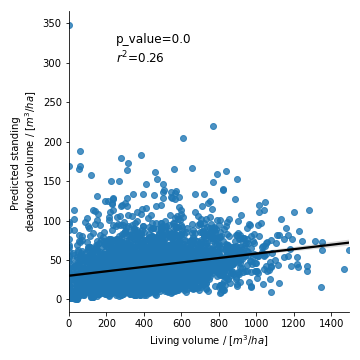

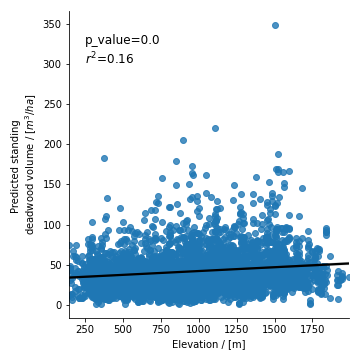

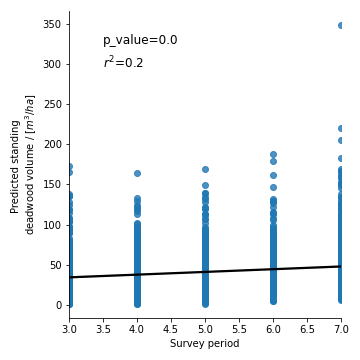

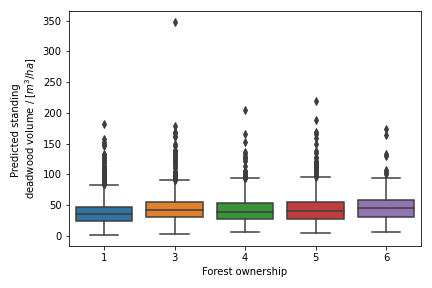

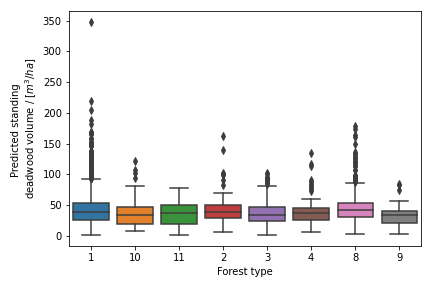

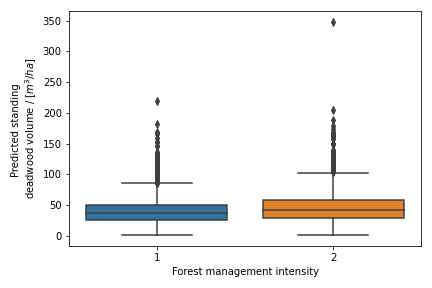


**Figure S4a-f.** Trend and magnitude between snag volume [m^3^ha^-1^] and the influencing predictors based on observations for (a) living volume stock [m^3^ha-^1^], (b) elevation [m], (c) NFI survey period (NFI3-NFI7) using linear regression plots and (d) forest ownership, (e) forest type and (f) forest management intensity using boxplots. Median values, 25% and 75% percentiles (boxes), min-max values (whiskers) and outliers are presented. A description of variables is presented in Table S1.


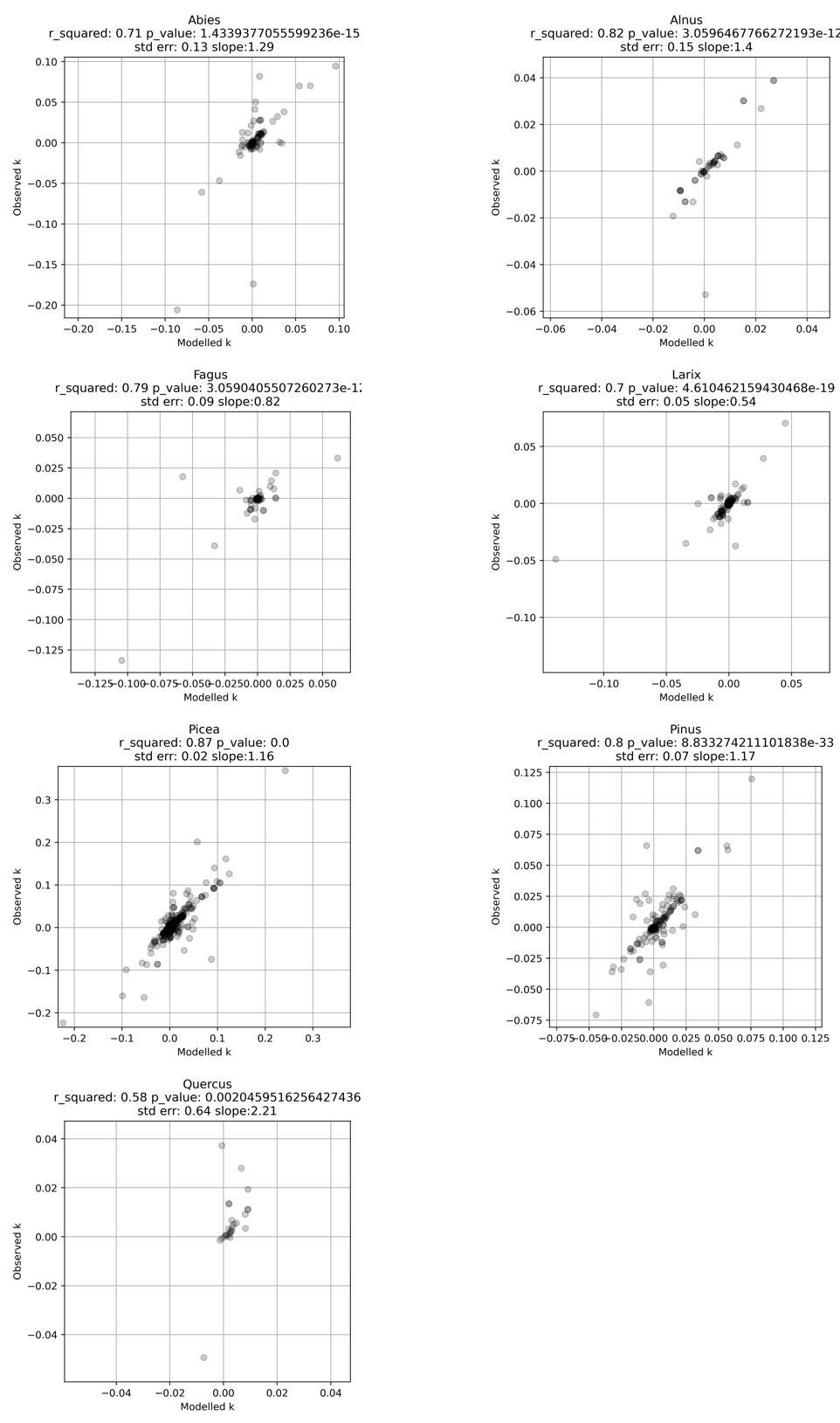


**Figure S5a.** Comparison of observed with modelled values for deadwood volume loss rate k_loss_ based on model a2 (negative and positive k-values, excluding broken trees) for seven tree genera.


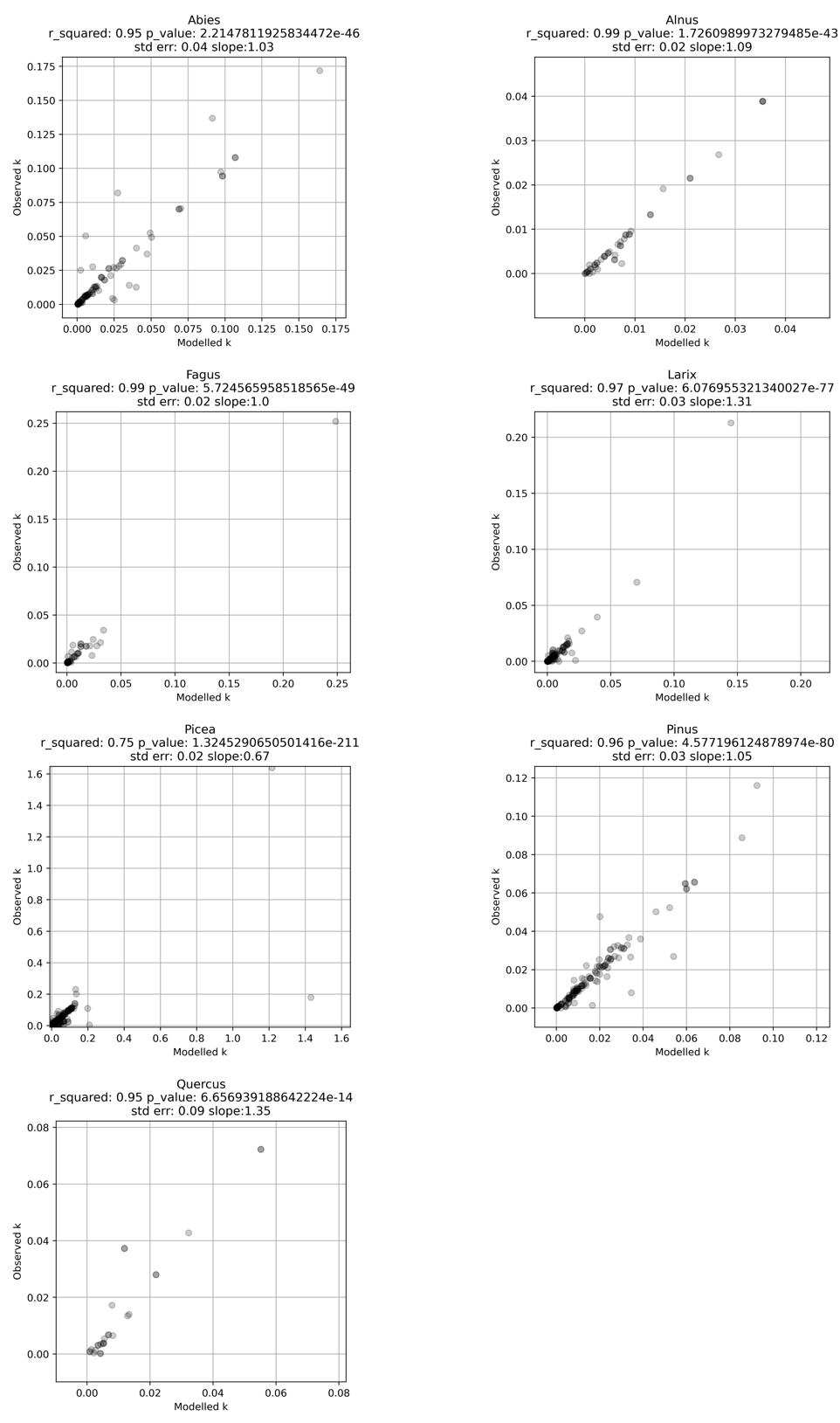


**Figure S5b.** Comparison of observed with modelled values for deadwood volume loss rate k_loss_ based on model b2 (only positive k-values, excluding broken trees) for seven tree genera.


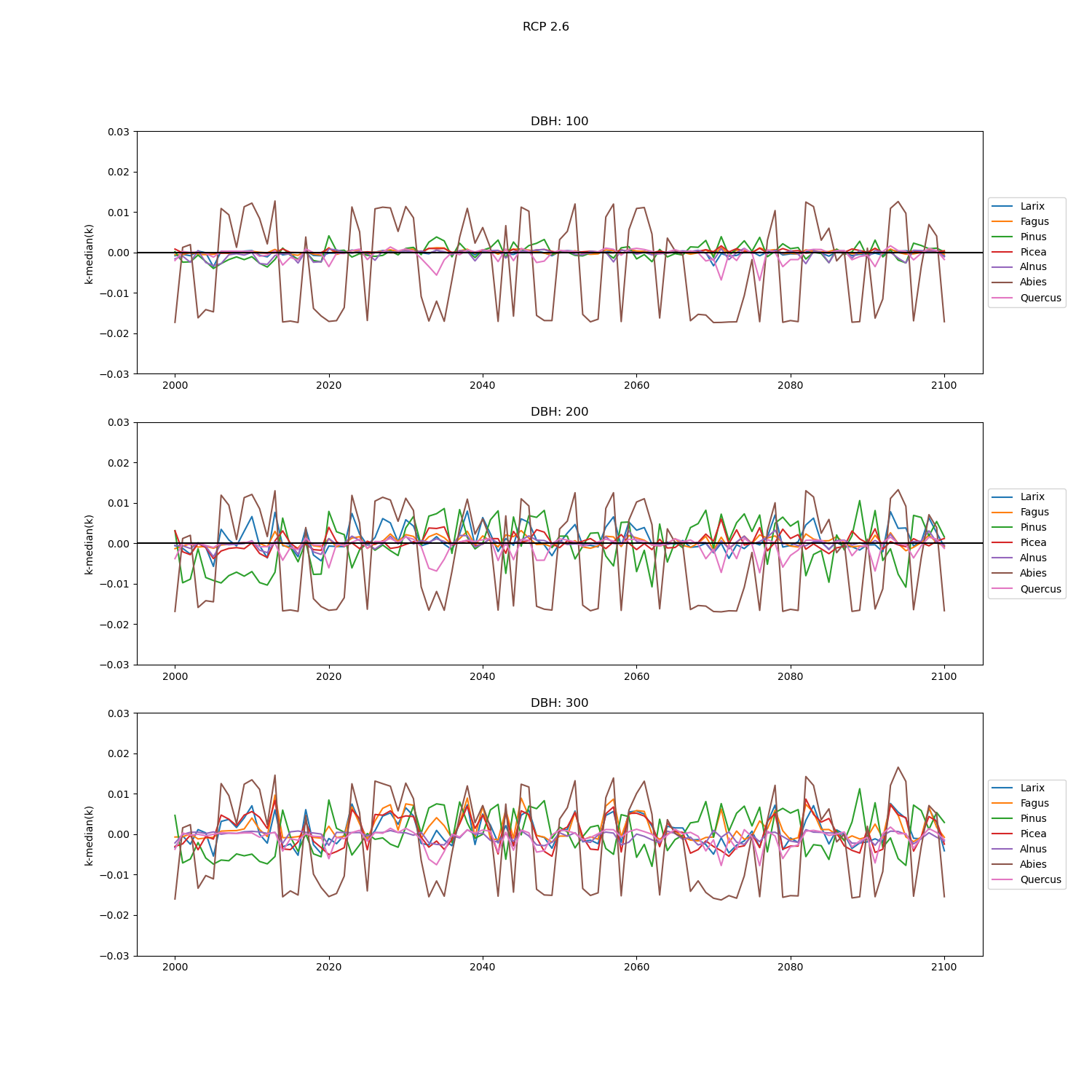


**
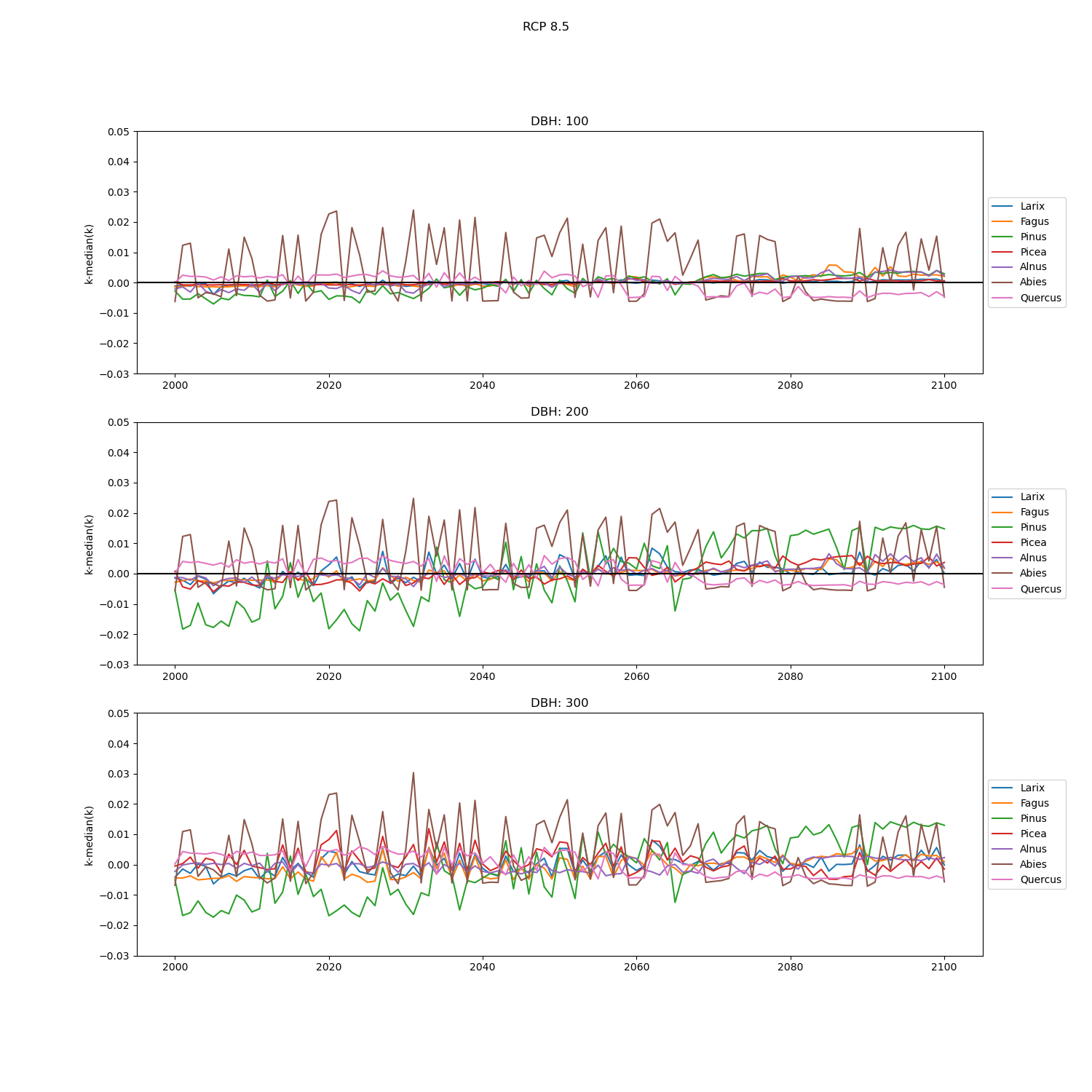
**

**Figure S6.** Prediction of snag volume loss rate k_loss_ [year^-1^] using **model a2** as baseline condition under climate change scenarios RCP2.6 and RCP8.5 for the seven investigated tree genera. Subplots refer to different tree diameters at breast height (dbh: 100, 200, 300 mm). To enable a comparison of volume loss among the investigated tree genera, the k_loss_-values are expressed as deviation from the respective median. The slopes and averages of the individual tree genera per diameter at breast height (dbh) are provided in Table S6.
