# Supplementary figures and images for: Dynamics of standing deadwood in Austrian forests under varying forest management and climatic conditions
Source: J Appl Ecol. 2023 Jan 24;60(4):696–713. doi: 10.1111/1365-2664.14359 (PMC10947403; doi:10.1111/1365-2664.14359)

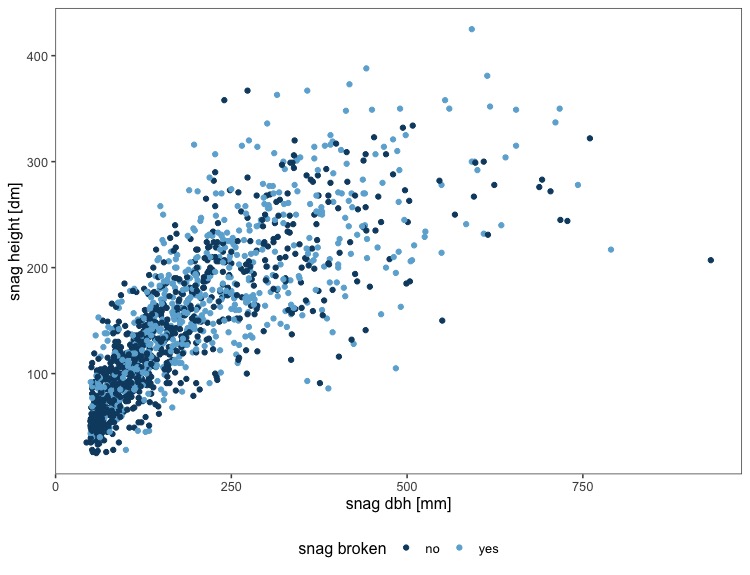

Supplement: Supplementary file 1 — Figure S1. Snag dbh (diameter at breast height [mm] and height [dm]) measurement values of the seventh Austrian NFI (National Forest Inventory) period (n = 1417). Light blue points indicate broken individuals (n = 601, 42.4%). Figure S2. Linear relationship (light blue line) between initial snag volume [m3] measured after tree death (initial volume) and the volume measured before snag fall (final volume) for the tree genera Abies (R2:0.99), Alnus (R2:0.95), Fagus (R2:0.98), Larix (R2:0.98), Picea (R2:0.94), Pinus (R2:0.95), Quercus (R2:0.99).The linear relationship is visualized for all tree genera with a 1:1 relationship (grey dashed line). Figure S3. Density estimates of the volume loss rate kloss [year−1] per tree genus. The estimate was performed with a Gaussian kernel and a bandwidth of 0.01. Figure S4a–f. Trend and magnitude between snag volume [m3ha−1] and the influencing predictors based on observations for (a) living volume stock [m3ha‐1], (b) elevation [m], (c) NFI survey period (NFI3‐NFI7) using linear regression plots and (d) forest ownership, (e) forest type and (f) forest management intensity using boxplots. Median values, 25% and 75% percentiles (boxes), min‐max values (whiskers) and outliers are presented. A description of variables is presented in Table S1. Figure S5a. Comparison of observed with modelled values for deadwood volume loss rate kloss based on model a2 (negative and positive k‐values, excluding broken trees) for seven tree genera. Figure S5b. Comparison of observed with modelled values for deadwood volume loss rate kloss based on model b2 (only positive k‐values, excluding broken trees) for seven tree genera. Figure S6. Prediction of snag volume loss rate kloss [year−1] using model a2 as baseline condition under climate change scenarios RCP2.6 and RCP8.5 for the seven investigated tree genera. Subplots refer to different tree diameters at breast height (dbh: 100, 200, 300 mm). To enable a comparison of volume loss among the investigated [file JPE-60-696-s001.zip › JPE_14359_Suppinfo_FigureS1.jpeg]

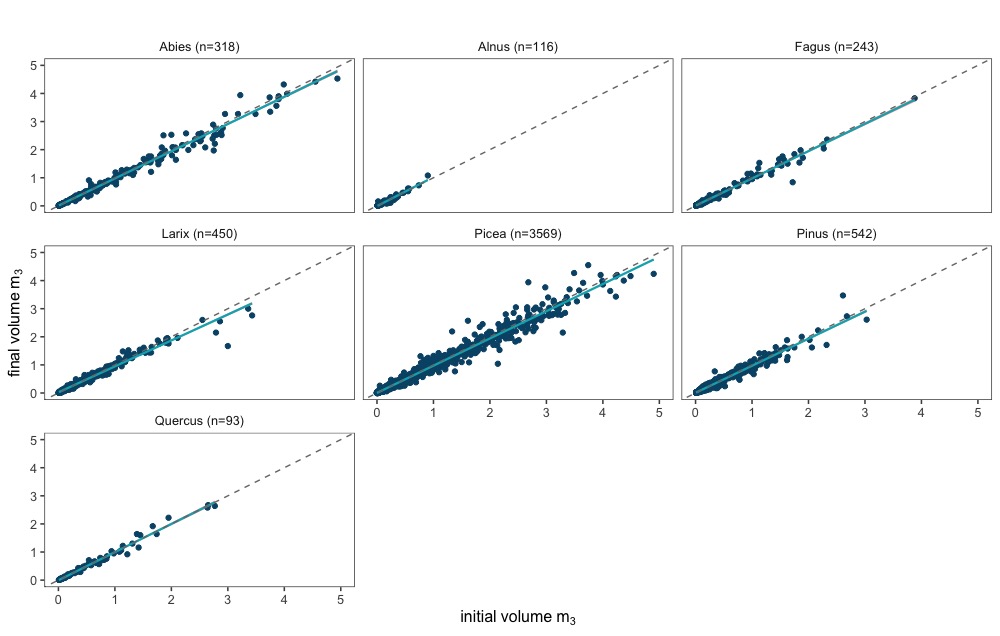

Supplement: Supplementary file 1 — Figure S1. Snag dbh (diameter at breast height [mm] and height [dm]) measurement values of the seventh Austrian NFI (National Forest Inventory) period (n = 1417). Light blue points indicate broken individuals (n = 601, 42.4%). Figure S2. Linear relationship (light blue line) between initial snag volume [m3] measured after tree death (initial volume) and the volume measured before snag fall (final volume) for the tree genera Abies (R2:0.99), Alnus (R2:0.95), Fagus (R2:0.98), Larix (R2:0.98), Picea (R2:0.94), Pinus (R2:0.95), Quercus (R2:0.99).The linear relationship is visualized for all tree genera with a 1:1 relationship (grey dashed line). Figure S3. Density estimates of the volume loss rate kloss [year−1] per tree genus. The estimate was performed with a Gaussian kernel and a bandwidth of 0.01. Figure S4a–f. Trend and magnitude between snag volume [m3ha−1] and the influencing predictors based on observations for (a) living volume stock [m3ha‐1], (b) elevation [m], (c) NFI survey period (NFI3‐NFI7) using linear regression plots and (d) forest ownership, (e) forest type and (f) forest management intensity using boxplots. Median values, 25% and 75% percentiles (boxes), min‐max values (whiskers) and outliers are presented. A description of variables is presented in Table S1. Figure S5a. Comparison of observed with modelled values for deadwood volume loss rate kloss based on model a2 (negative and positive k‐values, excluding broken trees) for seven tree genera. Figure S5b. Comparison of observed with modelled values for deadwood volume loss rate kloss based on model b2 (only positive k‐values, excluding broken trees) for seven tree genera. Figure S6. Prediction of snag volume loss rate kloss [year−1] using model a2 as baseline condition under climate change scenarios RCP2.6 and RCP8.5 for the seven investigated tree genera. Subplots refer to different tree diameters at breast height (dbh: 100, 200, 300 mm). To enable a comparison of volume loss among the investigated [file JPE-60-696-s001.zip › JPE_14359_Suppinfo_FigureS2.jpeg]

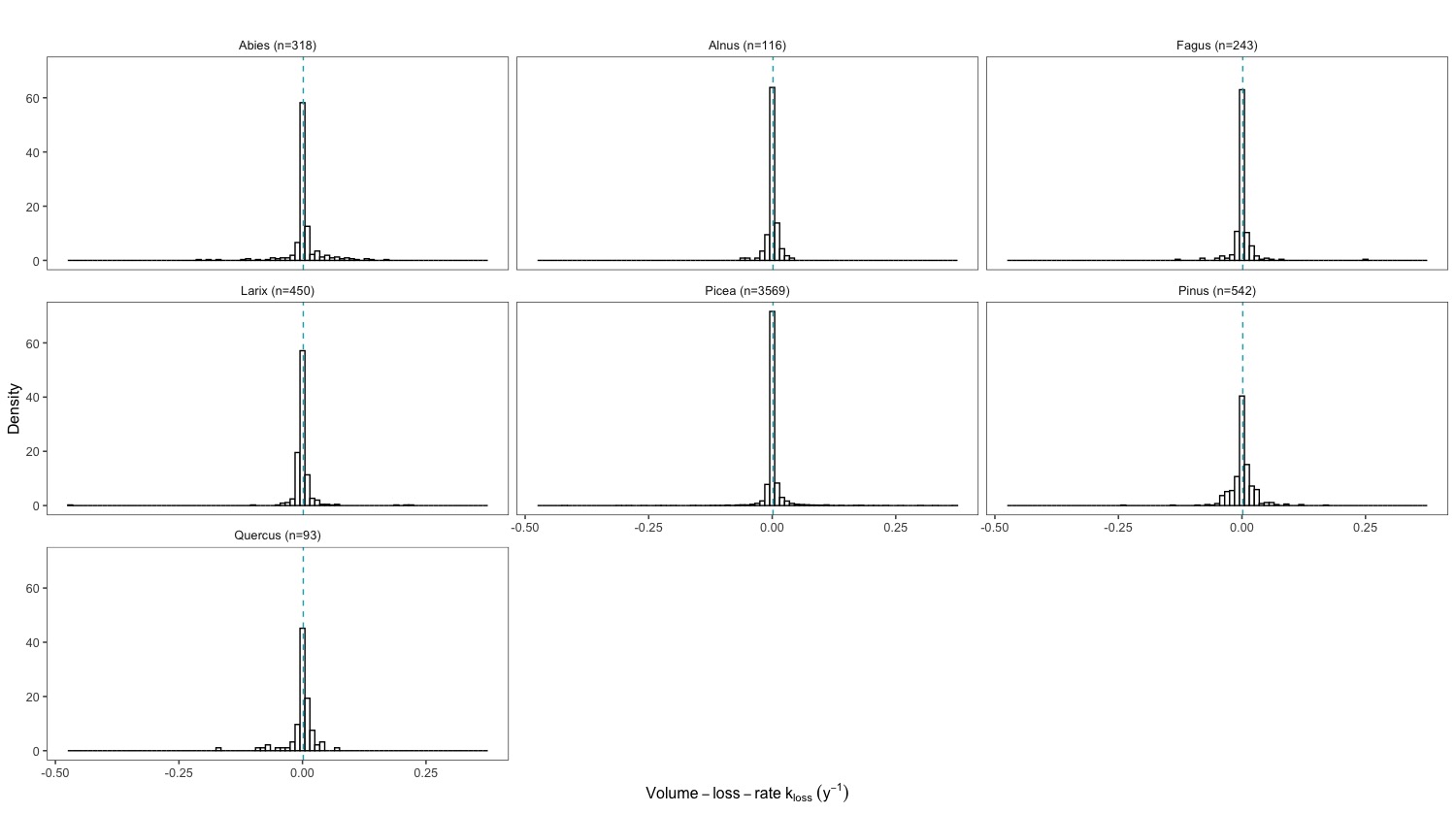

Supplement: Supplementary file 1 — Figure S1. Snag dbh (diameter at breast height [mm] and height [dm]) measurement values of the seventh Austrian NFI (National Forest Inventory) period (n = 1417). Light blue points indicate broken individuals (n = 601, 42.4%). Figure S2. Linear relationship (light blue line) between initial snag volume [m3] measured after tree death (initial volume) and the volume measured before snag fall (final volume) for the tree genera Abies (R2:0.99), Alnus (R2:0.95), Fagus (R2:0.98), Larix (R2:0.98), Picea (R2:0.94), Pinus (R2:0.95), Quercus (R2:0.99).The linear relationship is visualized for all tree genera with a 1:1 relationship (grey dashed line). Figure S3. Density estimates of the volume loss rate kloss [year−1] per tree genus. The estimate was performed with a Gaussian kernel and a bandwidth of 0.01. Figure S4a–f. Trend and magnitude between snag volume [m3ha−1] and the influencing predictors based on observations for (a) living volume stock [m3ha‐1], (b) elevation [m], (c) NFI survey period (NFI3‐NFI7) using linear regression plots and (d) forest ownership, (e) forest type and (f) forest management intensity using boxplots. Median values, 25% and 75% percentiles (boxes), min‐max values (whiskers) and outliers are presented. A description of variables is presented in Table S1. Figure S5a. Comparison of observed with modelled values for deadwood volume loss rate kloss based on model a2 (negative and positive k‐values, excluding broken trees) for seven tree genera. Figure S5b. Comparison of observed with modelled values for deadwood volume loss rate kloss based on model b2 (only positive k‐values, excluding broken trees) for seven tree genera. Figure S6. Prediction of snag volume loss rate kloss [year−1] using model a2 as baseline condition under climate change scenarios RCP2.6 and RCP8.5 for the seven investigated tree genera. Subplots refer to different tree diameters at breast height (dbh: 100, 200, 300 mm). To enable a comparison of volume loss among the investigated [file JPE-60-696-s001.zip › JPE_14359_Suppinfo_FigureS3.jpeg]

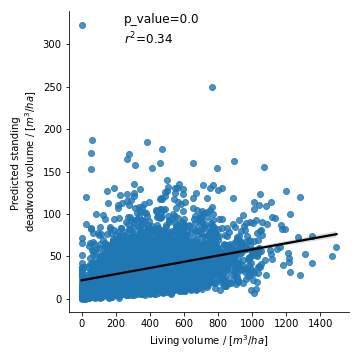

Supplement: Supplementary file 1 — Figure S1. Snag dbh (diameter at breast height [mm] and height [dm]) measurement values of the seventh Austrian NFI (National Forest Inventory) period (n = 1417). Light blue points indicate broken individuals (n = 601, 42.4%). Figure S2. Linear relationship (light blue line) between initial snag volume [m3] measured after tree death (initial volume) and the volume measured before snag fall (final volume) for the tree genera Abies (R2:0.99), Alnus (R2:0.95), Fagus (R2:0.98), Larix (R2:0.98), Picea (R2:0.94), Pinus (R2:0.95), Quercus (R2:0.99).The linear relationship is visualized for all tree genera with a 1:1 relationship (grey dashed line). Figure S3. Density estimates of the volume loss rate kloss [year−1] per tree genus. The estimate was performed with a Gaussian kernel and a bandwidth of 0.01. Figure S4a–f. Trend and magnitude between snag volume [m3ha−1] and the influencing predictors based on observations for (a) living volume stock [m3ha‐1], (b) elevation [m], (c) NFI survey period (NFI3‐NFI7) using linear regression plots and (d) forest ownership, (e) forest type and (f) forest management intensity using boxplots. Median values, 25% and 75% percentiles (boxes), min‐max values (whiskers) and outliers are presented. A description of variables is presented in Table S1. Figure S5a. Comparison of observed with modelled values for deadwood volume loss rate kloss based on model a2 (negative and positive k‐values, excluding broken trees) for seven tree genera. Figure S5b. Comparison of observed with modelled values for deadwood volume loss rate kloss based on model b2 (only positive k‐values, excluding broken trees) for seven tree genera. Figure S6. Prediction of snag volume loss rate kloss [year−1] using model a2 as baseline condition under climate change scenarios RCP2.6 and RCP8.5 for the seven investigated tree genera. Subplots refer to different tree diameters at breast height (dbh: 100, 200, 300 mm). To enable a comparison of volume loss among the investigated [file JPE-60-696-s001.zip › JPE_14359_Suppinfo_FigureS4a.png]

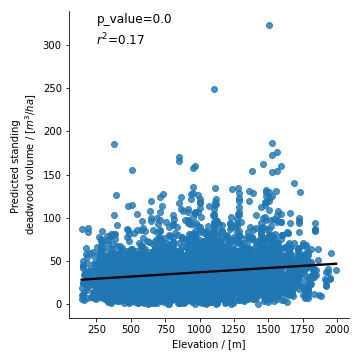

Supplement: Supplementary file 1 — Figure S1. Snag dbh (diameter at breast height [mm] and height [dm]) measurement values of the seventh Austrian NFI (National Forest Inventory) period (n = 1417). Light blue points indicate broken individuals (n = 601, 42.4%). Figure S2. Linear relationship (light blue line) between initial snag volume [m3] measured after tree death (initial volume) and the volume measured before snag fall (final volume) for the tree genera Abies (R2:0.99), Alnus (R2:0.95), Fagus (R2:0.98), Larix (R2:0.98), Picea (R2:0.94), Pinus (R2:0.95), Quercus (R2:0.99).The linear relationship is visualized for all tree genera with a 1:1 relationship (grey dashed line). Figure S3. Density estimates of the volume loss rate kloss [year−1] per tree genus. The estimate was performed with a Gaussian kernel and a bandwidth of 0.01. Figure S4a–f. Trend and magnitude between snag volume [m3ha−1] and the influencing predictors based on observations for (a) living volume stock [m3ha‐1], (b) elevation [m], (c) NFI survey period (NFI3‐NFI7) using linear regression plots and (d) forest ownership, (e) forest type and (f) forest management intensity using boxplots. Median values, 25% and 75% percentiles (boxes), min‐max values (whiskers) and outliers are presented. A description of variables is presented in Table S1. Figure S5a. Comparison of observed with modelled values for deadwood volume loss rate kloss based on model a2 (negative and positive k‐values, excluding broken trees) for seven tree genera. Figure S5b. Comparison of observed with modelled values for deadwood volume loss rate kloss based on model b2 (only positive k‐values, excluding broken trees) for seven tree genera. Figure S6. Prediction of snag volume loss rate kloss [year−1] using model a2 as baseline condition under climate change scenarios RCP2.6 and RCP8.5 for the seven investigated tree genera. Subplots refer to different tree diameters at breast height (dbh: 100, 200, 300 mm). To enable a comparison of volume loss among the investigated [file JPE-60-696-s001.zip › JPE_14359_Suppinfo_FigureS4b.png]

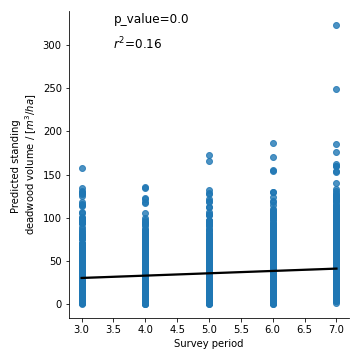

Supplement: Supplementary file 1 — Figure S1. Snag dbh (diameter at breast height [mm] and height [dm]) measurement values of the seventh Austrian NFI (National Forest Inventory) period (n = 1417). Light blue points indicate broken individuals (n = 601, 42.4%). Figure S2. Linear relationship (light blue line) between initial snag volume [m3] measured after tree death (initial volume) and the volume measured before snag fall (final volume) for the tree genera Abies (R2:0.99), Alnus (R2:0.95), Fagus (R2:0.98), Larix (R2:0.98), Picea (R2:0.94), Pinus (R2:0.95), Quercus (R2:0.99).The linear relationship is visualized for all tree genera with a 1:1 relationship (grey dashed line). Figure S3. Density estimates of the volume loss rate kloss [year−1] per tree genus. The estimate was performed with a Gaussian kernel and a bandwidth of 0.01. Figure S4a–f. Trend and magnitude between snag volume [m3ha−1] and the influencing predictors based on observations for (a) living volume stock [m3ha‐1], (b) elevation [m], (c) NFI survey period (NFI3‐NFI7) using linear regression plots and (d) forest ownership, (e) forest type and (f) forest management intensity using boxplots. Median values, 25% and 75% percentiles (boxes), min‐max values (whiskers) and outliers are presented. A description of variables is presented in Table S1. Figure S5a. Comparison of observed with modelled values for deadwood volume loss rate kloss based on model a2 (negative and positive k‐values, excluding broken trees) for seven tree genera. Figure S5b. Comparison of observed with modelled values for deadwood volume loss rate kloss based on model b2 (only positive k‐values, excluding broken trees) for seven tree genera. Figure S6. Prediction of snag volume loss rate kloss [year−1] using model a2 as baseline condition under climate change scenarios RCP2.6 and RCP8.5 for the seven investigated tree genera. Subplots refer to different tree diameters at breast height (dbh: 100, 200, 300 mm). To enable a comparison of volume loss among the investigated [file JPE-60-696-s001.zip › JPE_14359_Suppinfo_FigureS4c.png]

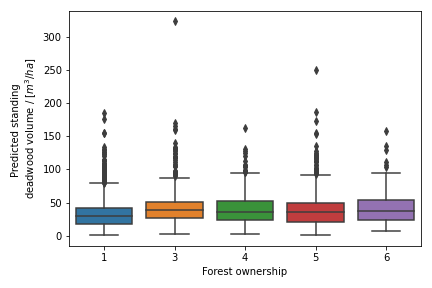

Supplement: Supplementary file 1 — Figure S1. Snag dbh (diameter at breast height [mm] and height [dm]) measurement values of the seventh Austrian NFI (National Forest Inventory) period (n = 1417). Light blue points indicate broken individuals (n = 601, 42.4%). Figure S2. Linear relationship (light blue line) between initial snag volume [m3] measured after tree death (initial volume) and the volume measured before snag fall (final volume) for the tree genera Abies (R2:0.99), Alnus (R2:0.95), Fagus (R2:0.98), Larix (R2:0.98), Picea (R2:0.94), Pinus (R2:0.95), Quercus (R2:0.99).The linear relationship is visualized for all tree genera with a 1:1 relationship (grey dashed line). Figure S3. Density estimates of the volume loss rate kloss [year−1] per tree genus. The estimate was performed with a Gaussian kernel and a bandwidth of 0.01. Figure S4a–f. Trend and magnitude between snag volume [m3ha−1] and the influencing predictors based on observations for (a) living volume stock [m3ha‐1], (b) elevation [m], (c) NFI survey period (NFI3‐NFI7) using linear regression plots and (d) forest ownership, (e) forest type and (f) forest management intensity using boxplots. Median values, 25% and 75% percentiles (boxes), min‐max values (whiskers) and outliers are presented. A description of variables is presented in Table S1. Figure S5a. Comparison of observed with modelled values for deadwood volume loss rate kloss based on model a2 (negative and positive k‐values, excluding broken trees) for seven tree genera. Figure S5b. Comparison of observed with modelled values for deadwood volume loss rate kloss based on model b2 (only positive k‐values, excluding broken trees) for seven tree genera. Figure S6. Prediction of snag volume loss rate kloss [year−1] using model a2 as baseline condition under climate change scenarios RCP2.6 and RCP8.5 for the seven investigated tree genera. Subplots refer to different tree diameters at breast height (dbh: 100, 200, 300 mm). To enable a comparison of volume loss among the investigated [file JPE-60-696-s001.zip › JPE_14359_Suppinfo_FigureS4d.png]

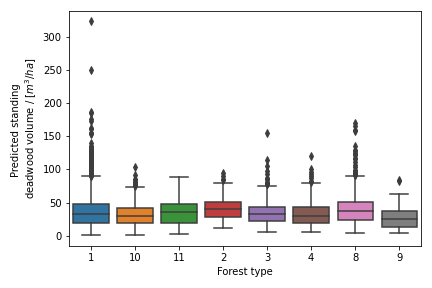

Supplement: Supplementary file 1 — Figure S1. Snag dbh (diameter at breast height [mm] and height [dm]) measurement values of the seventh Austrian NFI (National Forest Inventory) period (n = 1417). Light blue points indicate broken individuals (n = 601, 42.4%). Figure S2. Linear relationship (light blue line) between initial snag volume [m3] measured after tree death (initial volume) and the volume measured before snag fall (final volume) for the tree genera Abies (R2:0.99), Alnus (R2:0.95), Fagus (R2:0.98), Larix (R2:0.98), Picea (R2:0.94), Pinus (R2:0.95), Quercus (R2:0.99).The linear relationship is visualized for all tree genera with a 1:1 relationship (grey dashed line). Figure S3. Density estimates of the volume loss rate kloss [year−1] per tree genus. The estimate was performed with a Gaussian kernel and a bandwidth of 0.01. Figure S4a–f. Trend and magnitude between snag volume [m3ha−1] and the influencing predictors based on observations for (a) living volume stock [m3ha‐1], (b) elevation [m], (c) NFI survey period (NFI3‐NFI7) using linear regression plots and (d) forest ownership, (e) forest type and (f) forest management intensity using boxplots. Median values, 25% and 75% percentiles (boxes), min‐max values (whiskers) and outliers are presented. A description of variables is presented in Table S1. Figure S5a. Comparison of observed with modelled values for deadwood volume loss rate kloss based on model a2 (negative and positive k‐values, excluding broken trees) for seven tree genera. Figure S5b. Comparison of observed with modelled values for deadwood volume loss rate kloss based on model b2 (only positive k‐values, excluding broken trees) for seven tree genera. Figure S6. Prediction of snag volume loss rate kloss [year−1] using model a2 as baseline condition under climate change scenarios RCP2.6 and RCP8.5 for the seven investigated tree genera. Subplots refer to different tree diameters at breast height (dbh: 100, 200, 300 mm). To enable a comparison of volume loss among the investigated [file JPE-60-696-s001.zip › JPE_14359_Suppinfo_FigureS4e.png]

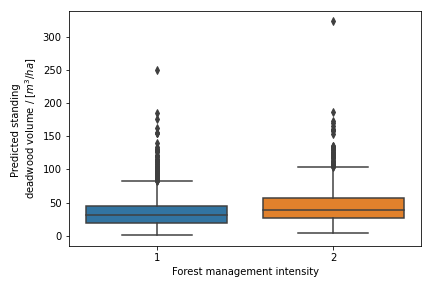

Supplement: Supplementary file 1 — Figure S1. Snag dbh (diameter at breast height [mm] and height [dm]) measurement values of the seventh Austrian NFI (National Forest Inventory) period (n = 1417). Light blue points indicate broken individuals (n = 601, 42.4%). Figure S2. Linear relationship (light blue line) between initial snag volume [m3] measured after tree death (initial volume) and the volume measured before snag fall (final volume) for the tree genera Abies (R2:0.99), Alnus (R2:0.95), Fagus (R2:0.98), Larix (R2:0.98), Picea (R2:0.94), Pinus (R2:0.95), Quercus (R2:0.99).The linear relationship is visualized for all tree genera with a 1:1 relationship (grey dashed line). Figure S3. Density estimates of the volume loss rate kloss [year−1] per tree genus. The estimate was performed with a Gaussian kernel and a bandwidth of 0.01. Figure S4a–f. Trend and magnitude between snag volume [m3ha−1] and the influencing predictors based on observations for (a) living volume stock [m3ha‐1], (b) elevation [m], (c) NFI survey period (NFI3‐NFI7) using linear regression plots and (d) forest ownership, (e) forest type and (f) forest management intensity using boxplots. Median values, 25% and 75% percentiles (boxes), min‐max values (whiskers) and outliers are presented. A description of variables is presented in Table S1. Figure S5a. Comparison of observed with modelled values for deadwood volume loss rate kloss based on model a2 (negative and positive k‐values, excluding broken trees) for seven tree genera. Figure S5b. Comparison of observed with modelled values for deadwood volume loss rate kloss based on model b2 (only positive k‐values, excluding broken trees) for seven tree genera. Figure S6. Prediction of snag volume loss rate kloss [year−1] using model a2 as baseline condition under climate change scenarios RCP2.6 and RCP8.5 for the seven investigated tree genera. Subplots refer to different tree diameters at breast height (dbh: 100, 200, 300 mm). To enable a comparison of volume loss among the investigated [file JPE-60-696-s001.zip › JPE_14359_Suppinfo_FigureS4f.png]

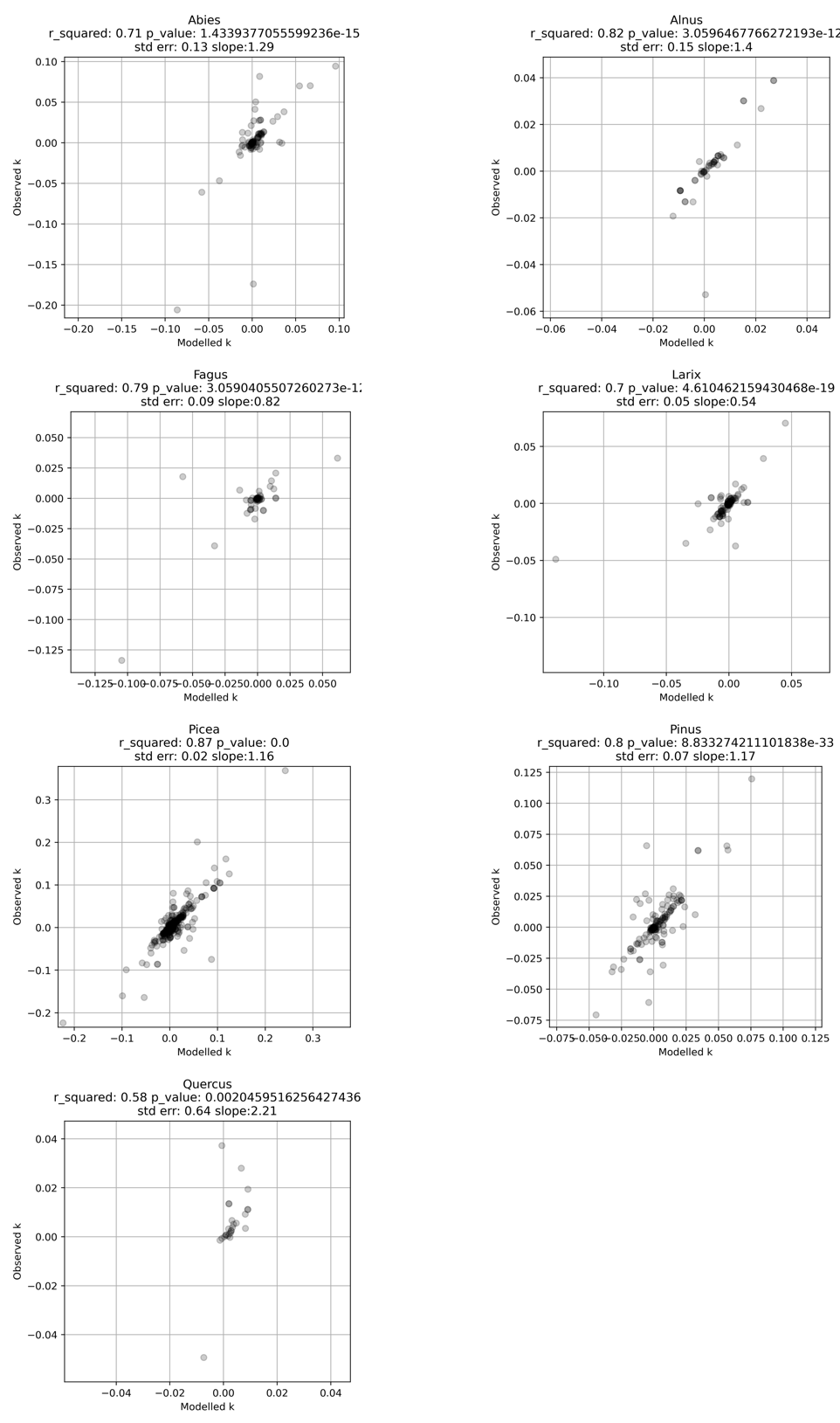

Supplement: Supplementary file 1 — Figure S1. Snag dbh (diameter at breast height [mm] and height [dm]) measurement values of the seventh Austrian NFI (National Forest Inventory) period (n = 1417). Light blue points indicate broken individuals (n = 601, 42.4%). Figure S2. Linear relationship (light blue line) between initial snag volume [m3] measured after tree death (initial volume) and the volume measured before snag fall (final volume) for the tree genera Abies (R2:0.99), Alnus (R2:0.95), Fagus (R2:0.98), Larix (R2:0.98), Picea (R2:0.94), Pinus (R2:0.95), Quercus (R2:0.99).The linear relationship is visualized for all tree genera with a 1:1 relationship (grey dashed line). Figure S3. Density estimates of the volume loss rate kloss [year−1] per tree genus. The estimate was performed with a Gaussian kernel and a bandwidth of 0.01. Figure S4a–f. Trend and magnitude between snag volume [m3ha−1] and the influencing predictors based on observations for (a) living volume stock [m3ha‐1], (b) elevation [m], (c) NFI survey period (NFI3‐NFI7) using linear regression plots and (d) forest ownership, (e) forest type and (f) forest management intensity using boxplots. Median values, 25% and 75% percentiles (boxes), min‐max values (whiskers) and outliers are presented. A description of variables is presented in Table S1. Figure S5a. Comparison of observed with modelled values for deadwood volume loss rate kloss based on model a2 (negative and positive k‐values, excluding broken trees) for seven tree genera. Figure S5b. Comparison of observed with modelled values for deadwood volume loss rate kloss based on model b2 (only positive k‐values, excluding broken trees) for seven tree genera. Figure S6. Prediction of snag volume loss rate kloss [year−1] using model a2 as baseline condition under climate change scenarios RCP2.6 and RCP8.5 for the seven investigated tree genera. Subplots refer to different tree diameters at breast height (dbh: 100, 200, 300 mm). To enable a comparison of volume loss among the investigated [file JPE-60-696-s001.zip › JPE_14359_Suppinfo_FigureS5a.png]

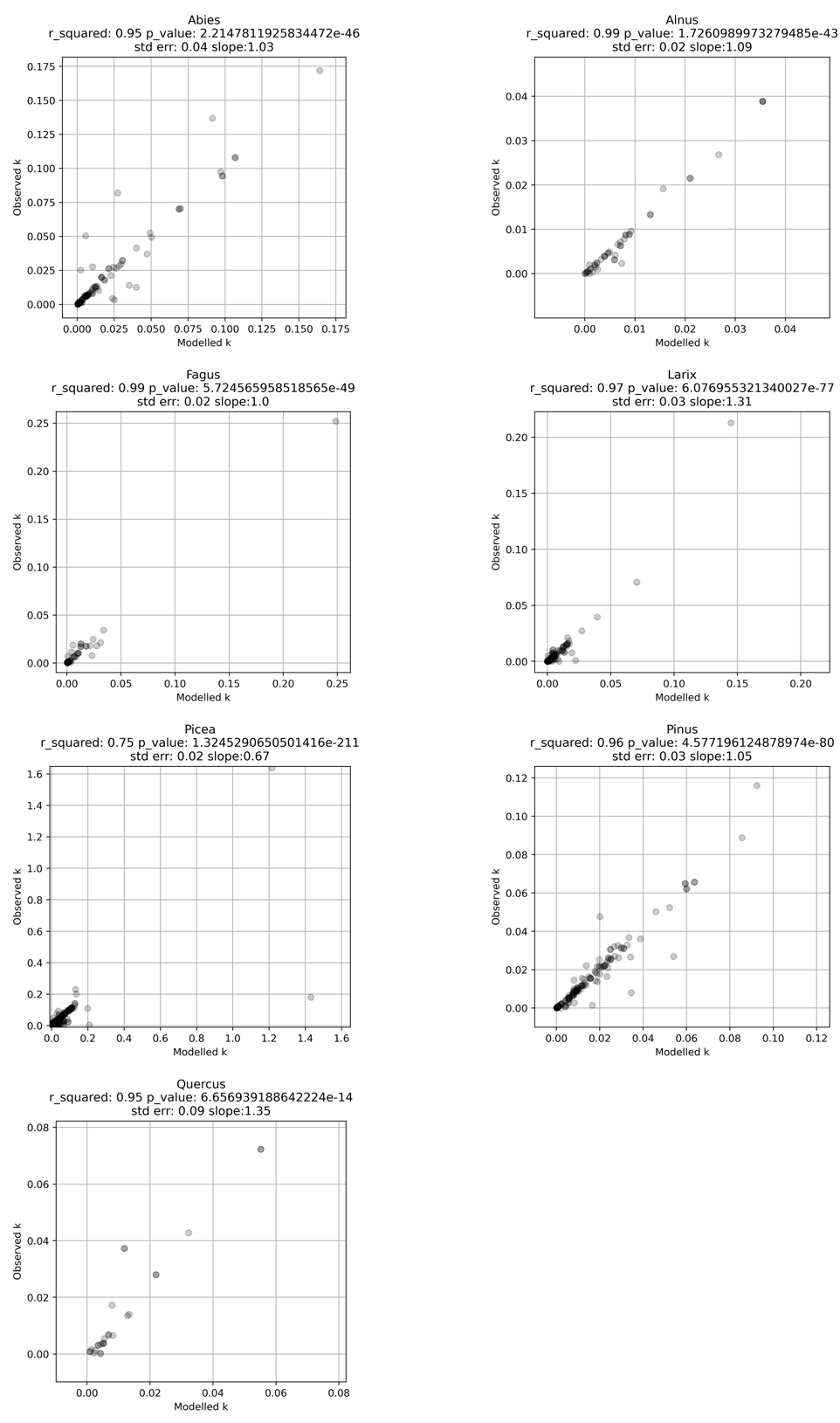

Supplement: Supplementary file 1 — Figure S1. Snag dbh (diameter at breast height [mm] and height [dm]) measurement values of the seventh Austrian NFI (National Forest Inventory) period (n = 1417). Light blue points indicate broken individuals (n = 601, 42.4%). Figure S2. Linear relationship (light blue line) between initial snag volume [m3] measured after tree death (initial volume) and the volume measured before snag fall (final volume) for the tree genera Abies (R2:0.99), Alnus (R2:0.95), Fagus (R2:0.98), Larix (R2:0.98), Picea (R2:0.94), Pinus (R2:0.95), Quercus (R2:0.99).The linear relationship is visualized for all tree genera with a 1:1 relationship (grey dashed line). Figure S3. Density estimates of the volume loss rate kloss [year−1] per tree genus. The estimate was performed with a Gaussian kernel and a bandwidth of 0.01. Figure S4a–f. Trend and magnitude between snag volume [m3ha−1] and the influencing predictors based on observations for (a) living volume stock [m3ha‐1], (b) elevation [m], (c) NFI survey period (NFI3‐NFI7) using linear regression plots and (d) forest ownership, (e) forest type and (f) forest management intensity using boxplots. Median values, 25% and 75% percentiles (boxes), min‐max values (whiskers) and outliers are presented. A description of variables is presented in Table S1. Figure S5a. Comparison of observed with modelled values for deadwood volume loss rate kloss based on model a2 (negative and positive k‐values, excluding broken trees) for seven tree genera. Figure S5b. Comparison of observed with modelled values for deadwood volume loss rate kloss based on model b2 (only positive k‐values, excluding broken trees) for seven tree genera. Figure S6. Prediction of snag volume loss rate kloss [year−1] using model a2 as baseline condition under climate change scenarios RCP2.6 and RCP8.5 for the seven investigated tree genera. Subplots refer to different tree diameters at breast height (dbh: 100, 200, 300 mm). To enable a comparison of volume loss among the investigated [file JPE-60-696-s001.zip › JPE_14359_Suppinfo_FigureS5b.png]

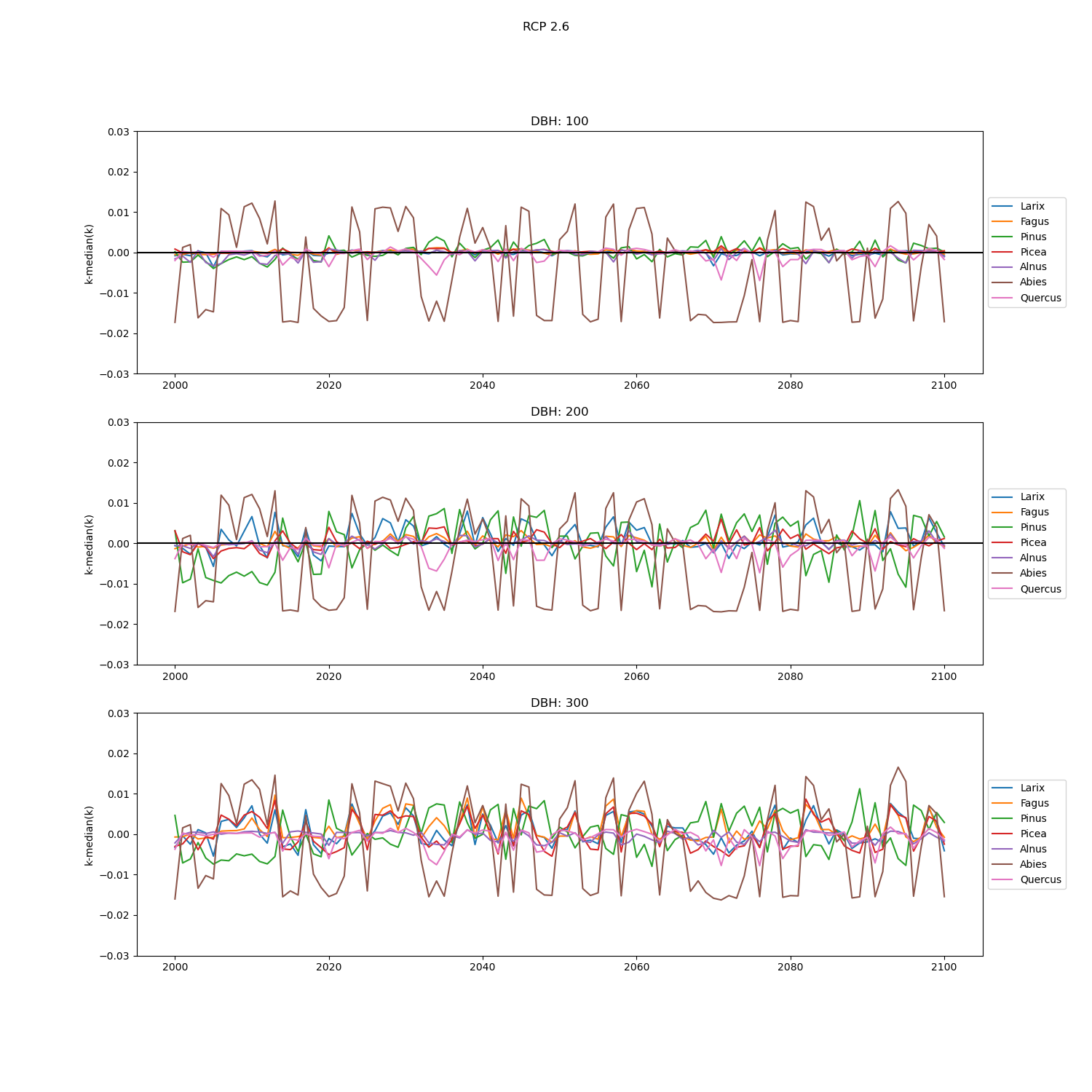

Supplement: Supplementary file 1 — Figure S1. Snag dbh (diameter at breast height [mm] and height [dm]) measurement values of the seventh Austrian NFI (National Forest Inventory) period (n = 1417). Light blue points indicate broken individuals (n = 601, 42.4%). Figure S2. Linear relationship (light blue line) between initial snag volume [m3] measured after tree death (initial volume) and the volume measured before snag fall (final volume) for the tree genera Abies (R2:0.99), Alnus (R2:0.95), Fagus (R2:0.98), Larix (R2:0.98), Picea (R2:0.94), Pinus (R2:0.95), Quercus (R2:0.99).The linear relationship is visualized for all tree genera with a 1:1 relationship (grey dashed line). Figure S3. Density estimates of the volume loss rate kloss [year−1] per tree genus. The estimate was performed with a Gaussian kernel and a bandwidth of 0.01. Figure S4a–f. Trend and magnitude between snag volume [m3ha−1] and the influencing predictors based on observations for (a) living volume stock [m3ha‐1], (b) elevation [m], (c) NFI survey period (NFI3‐NFI7) using linear regression plots and (d) forest ownership, (e) forest type and (f) forest management intensity using boxplots. Median values, 25% and 75% percentiles (boxes), min‐max values (whiskers) and outliers are presented. A description of variables is presented in Table S1. Figure S5a. Comparison of observed with modelled values for deadwood volume loss rate kloss based on model a2 (negative and positive k‐values, excluding broken trees) for seven tree genera. Figure S5b. Comparison of observed with modelled values for deadwood volume loss rate kloss based on model b2 (only positive k‐values, excluding broken trees) for seven tree genera. Figure S6. Prediction of snag volume loss rate kloss [year−1] using model a2 as baseline condition under climate change scenarios RCP2.6 and RCP8.5 for the seven investigated tree genera. Subplots refer to different tree diameters at breast height (dbh: 100, 200, 300 mm). To enable a comparison of volume loss among the investigated [file JPE-60-696-s001.zip › JPE_14359_Suppinfo_FigureS6_RCP2.6.png]

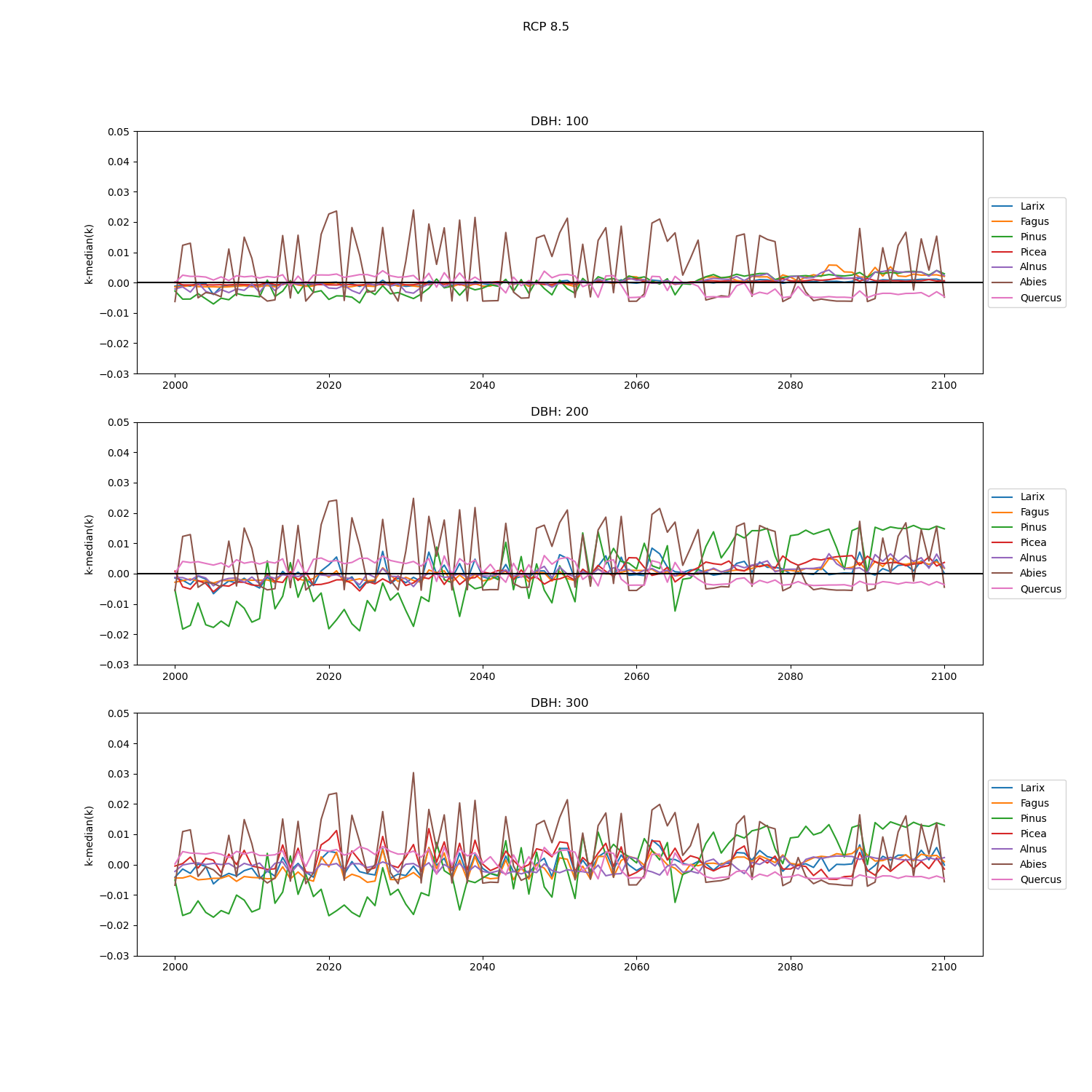

Supplement: Supplementary file 1 — Figure S1. Snag dbh (diameter at breast height [mm] and height [dm]) measurement values of the seventh Austrian NFI (National Forest Inventory) period (n = 1417). Light blue points indicate broken individuals (n = 601, 42.4%). Figure S2. Linear relationship (light blue line) between initial snag volume [m3] measured after tree death (initial volume) and the volume measured before snag fall (final volume) for the tree genera Abies (R2:0.99), Alnus (R2:0.95), Fagus (R2:0.98), Larix (R2:0.98), Picea (R2:0.94), Pinus (R2:0.95), Quercus (R2:0.99).The linear relationship is visualized for all tree genera with a 1:1 relationship (grey dashed line). Figure S3. Density estimates of the volume loss rate kloss [year−1] per tree genus. The estimate was performed with a Gaussian kernel and a bandwidth of 0.01. Figure S4a–f. Trend and magnitude between snag volume [m3ha−1] and the influencing predictors based on observations for (a) living volume stock [m3ha‐1], (b) elevation [m], (c) NFI survey period (NFI3‐NFI7) using linear regression plots and (d) forest ownership, (e) forest type and (f) forest management intensity using boxplots. Median values, 25% and 75% percentiles (boxes), min‐max values (whiskers) and outliers are presented. A description of variables is presented in Table S1. Figure S5a. Comparison of observed with modelled values for deadwood volume loss rate kloss based on model a2 (negative and positive k‐values, excluding broken trees) for seven tree genera. Figure S5b. Comparison of observed with modelled values for deadwood volume loss rate kloss based on model b2 (only positive k‐values, excluding broken trees) for seven tree genera. Figure S6. Prediction of snag volume loss rate kloss [year−1] using model a2 as baseline condition under climate change scenarios RCP2.6 and RCP8.5 for the seven investigated tree genera. Subplots refer to different tree diameters at breast height (dbh: 100, 200, 300 mm). To enable a comparison of volume loss among the investigated [file JPE-60-696-s001.zip › JPE_14359_Suppinfo_FigureS6_RCP8.5.png]
